# Limited Added Effect of Proline‐Rich P6 Peptide to Cross‐Linked Hyaluronic Acid After Partial Lateral Meniscectomy in Sheep

**DOI:** 10.1002/smmd.70048

**Published:** 2026-08-24

**Authors:** Augustine Mark Saiz, Reem Jamous, Sabine Stötzel, Maryam Rahmati, Leonard Volrath Holmboe, Soren David Johnson, Øystein Øvrebø, Angela De Lauretis, Thaqif El Khassawna, Liebert P. Nogueira, Maria Permuy, Fernando Muñoz, Antonio Gonzalez Cantalapiedra, Carlotta De Luca, Ståle Petter Lyngstadaas, Håvard Jostein Haugen

**Affiliations:** ^1^ Department of Biomaterials Institute of Clinical Dentistry University of Oslo Oslo Norway; ^2^ Department of Orthopedic Surgery UC Davis Health Sacramento California USA; ^3^ Experimental Trauma Surgery Faculty of Medicine Justus Liebig University Giessen Giessen Germany; ^4^ Regenarti AS Oslo Norway; ^5^ Faculty of Pharmacy University of Jordan Amman Jordan; ^6^ Oral Research Laboratory Institute of Clinical Dentistry University of Oslo Oslo Norway; ^7^ Facultad de Veterinaria Universidade de Santiago de Compostela Campus Universitario Lugo Spain; ^8^ iBoneLab S.L. Lugo Spain; ^9^ Department of Biomedical and Dental Sciences and Morphofunctional Imaging University of Messina Messina Italy

**Keywords:** hyaluronic acid, injectable hydrogel, meniscal injury, osteochondral biomaterials, post‐traumatic osteoarthritis, proline‐rich peptide, sheep

## Abstract

This study evaluates whether a proline‐rich P6 peptide provides added benefit to a cross‐linked hyaluronic acid (HA) hydrogel after partial lateral meniscectomy‐associated osteoarthritis‐like change in sheep. The corrected four‐group framework includes Intact no‐surgery/no‐injection reference knees (*n* = 7) and 16 surgically injured knees from eight sheep assigned to Mnx (meniscectomy only, *n* = 4), Mnx + HA (*n* = 6), or Mnx + HA + P6 (*n* = 6). Mnx + HA and Mnx + HA + P6 knees received intra‐articular injections at Weeks 0, 1, 2, and 4. Osteochondral tissues were evaluated at Week 12 by microCT, H&E scoring, and histochemical/immunohistochemical image analysis. Endpoint‐specific animal‐ or knee‐level models were used, with sheep identity included as a random effect when estimable. HA‐based treatment was associated with favorable trabecular microCT patterns after meniscectomy. However, the primary Mnx + HA + P6 versus Mnx + HA contrast was not significant for percent trabecular bone volume/BV/TV (+1.52 percentage points; 95% CI −2.22 to 5.26; *p* = 0.427). Histology and IHC/histochemical findings showed selected exploratory tissue‐pattern signals, including collagen‐associated matrix staining, but did not establish consistent peptide‐specific superiority. These findings support cross‐linked HA as an intra‐articular biomaterial platform while defining requirements for confirmatory HA‐peptide hydrogel studies.

## Key Points

- The corrected analysis separates Intact, Mnx, Mnx + HA, and Mnx + HA + P6 groups and uses endpoint‐specific animal/knee‐level inference.
- HA‐based treatment was associated with favorable trabecular microCT patterns after meniscectomy, but Mnx + HA + P6 did not show consistent superiority over Mnx + HA.
- Histology and IHC/histochemical findings provide exploratory tissue‐pattern context but not confirmatory evidence of peptide‐specific efficacy.

## Graphical abstract

After partial lateral meniscectomy in sheep, cross‐linked hyaluronic acid‐based treatment was associated with favorable trabecular microCT patterns, whereas adding the proline‐rich P6 peptide did not show consistent benefit over cross‐linked HA. Exploratory matrix staining signals define priorities for confirmatory HA–peptide hydrogel studies.

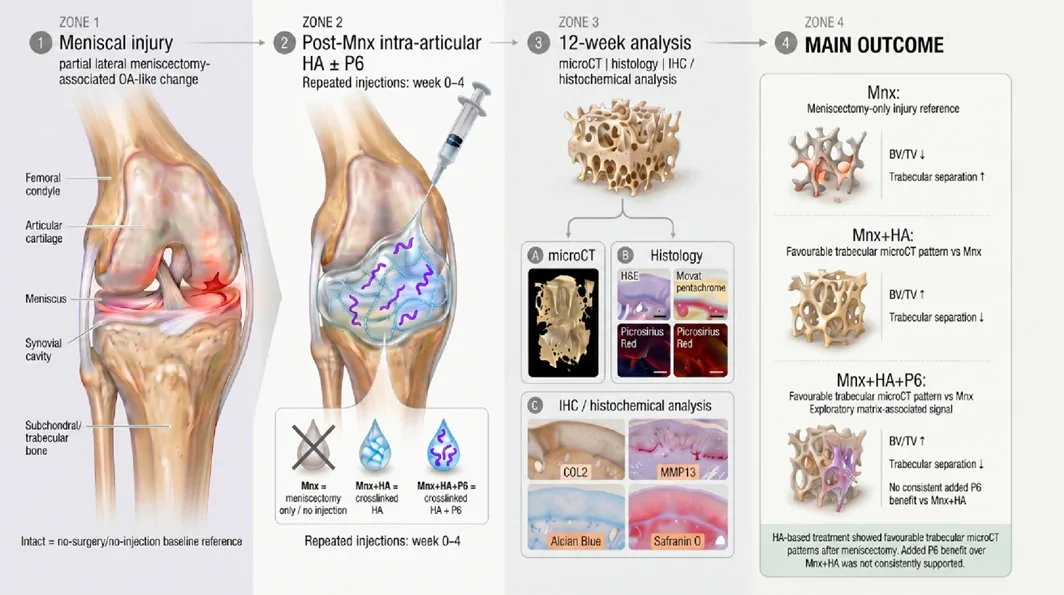

## 1 Introduction

Knee osteoarthritis (OA) is a major cause of pain, reduced mobility, and disability, and its burden continues to rise with aging populations, obesity, and joint injury [1, 2, 3]. It is no longer useful to describe OA as passive cartilage wear. The disease involves the whole joint, including articular cartilage, synovium, meniscus, subchondral bone, osteophytes, ligaments, periarticular tissues, and nociceptive pathways [4, 5, 6]. This whole‐joint view is particularly relevant in post‐traumatic osteoarthritis (PTOA), where a mechanical insult can trigger inflammation, altered joint mechanics, matrix degradation, and osteochondral remodeling [7, 8, 9].

Meniscal injury is one of the most clinically relevant triggers of knee PTOA. Menisci distribute load, improve joint congruity, contribute to lubrication, and protect cartilage from focal mechanical overload [10, 11, 12, 13]. When meniscal tissue is torn, destabilized, or removed, contact mechanics change, and the joint becomes prone to cartilage matrix loss, synovial activation, osteophyte formation, and subchondral bone adaptation [7, 10]. Ovine meniscal injury and meniscectomy models reproduce several early OA‐like features, including cartilage degeneration and joint effusion, and are useful for testing intra‐articular biomaterials in a mechanically loaded large joint [14, 15, 16, 17].

Synovial pathology is also part of this model and should not be treated as secondary to cartilage loss. In ovine meniscectomy models, synovial change is characterized mainly by subintimal fibrosis and hypervascularity, with more modest inflammatory‐cell infiltration [18, 19]. This matters for HA‐based materials because HA may influence not only lubrication and cartilage surface mechanics but also synovial remodeling and inflammatory signaling. Previous ovine work has shown that intra‐articular HA can reduce synovial vascularity and fibrosis‐related pathology after meniscectomy [18, 19].

Hyaluronic acid (HA) is an endogenous, non‐sulfated glycosaminoglycan that contributes to the viscoelastic properties of synovial fluid and the pericellular matrix of articular cartilage. In OA, the concentration and molecular‐weight distribution of endogenous HA are altered, with consequences for lubrication, shock absorption, and joint biochemical signaling [20, 21]. Although intra‐articular HA was introduced primarily as a viscosupplement, its proposed actions are broader and include CD44‐mediated signaling, modulation of catabolic enzymes and inflammatory mediators, proteoglycan synthesis, and nociceptive pathways [20, 22].

One weakness in the HA literature is that HA is often discussed as a single therapeutic class. This is chemically and biologically imprecise. HA formulations differ in molecular weight, concentration, source, crosslinking, degradation, rheology, and intra‐articular residence time [21, 23, 24, 25]. These material parameters can change both mechanical performance and biological response. Preclinical work supports this point: in a rat PTOA model, cross‐linked high‐molecular‐weight HA produced more sustained functional recovery, better cartilage preservation, higher type II collagen signal, and lower expression of catabolic and inflammatory markers than linear HA [26]. HA‐based intra‐articular treatments should therefore be evaluated as engineered biomaterials with defined structure‐function properties, not as interchangeable viscosupplements.

Peptide‐functionalized HA hydrogels offer a route to endow an injectable HA carrier with biological function [27, 28]. In such systems, HA provides a hydrated extracellular‐matrix‐relevant phase, while the peptide may affect cell‐material signaling, protein interactions, supramolecular organization, local retention, enzymatic susceptibility, or inflammatory crosstalk [27, 29, 30]. The practical challenge is not only bioactivity. In a mechanically active joint, an injectable material must also be deliverable, well‐tolerated, sufficiently retained, and compatible with local tissue remodeling [31, 32].

The P6 peptide belongs to a family of proline‐rich, intrinsically disordered peptides inspired by amelogenin and other non‐collagenous proteins involved in mineralized tissue formation [33, 34, 35, 36, 37]. Amelogenin‐related biology has also been linked to joint homeostasis, and recombinant amelogenin has shown repair‐associated effects in preclinical cartilage and osteochondral models [36, 37]. Previous work has shown that related peptides can influence wound healing, osteogenic differentiation, mineral nucleation, and bone formation [33, 34, 35]. P6 has also shown an immunomodulatory profile when incorporated into cross‐linked HA gels in an early soft‐tissue wound‐healing model, including reduced TNF‐α staining and reduced CD80‐associated inflammatory macrophage signaling [38].

The desired effect in the knee is not uncontrolled mineralization. In articular cartilage, excessive mineralizing activity could be undesirable. The more relevant question is whether peptide enrichment can modulate the early osteochondral and inflammatory environment without producing adverse tissue responses. The present study, therefore, evaluated HA and HA + P6 after partial lateral meniscectomy–associated OA‐like changes in sheep. The primary biological contrast was Mnx + HA + P6 versus Mnx + HA, because this directly tests whether the peptide adds value beyond the HA carrier. MicroCT of the osteochondral unit served as the principal structural readout, with histology and image‐based staining as supportive tissue‐level outcomes.

## 2 Methods

### 2.1 Study Design and Treatments

This controlled large‐animal pilot used a corrected four‐group knee‐level framework: Intact, partial lateral meniscectomy (Mnx), partial lateral meniscectomy and hyaluronic acid (Mnx + HA), and partial lateral meniscectomy and hyaluronic acid + synthetic peptide P6 (Mnx + HA + P6) (Table 1). Intact knees had no surgery and no intra‐articular injection and served as a no‐surgery/no‐injection baseline reference tissue. Mnx knees underwent surgically induced partial lateral meniscectomy/lateral hemidiscectomy but received no intra‐articular HA, HA + P6, saline, or vehicle injection. This group, therefore, served as the meniscectomy‐only injury reference and should not be interpreted as a sham‐operated or vehicle‐injected control.

**TABLE 1:** Animal/knee allocation and group sizes.

Group	Surgical procedure	Intra‐articular treatment	Role in analysis
Intact (*n* = 7 knees)	No meniscectomy and no surgical induction of OA‐like change.	No intra‐articular HA, HA + P6, saline, or vehicle injection.	No‐surgery/no‐injection baseline reference tissue.
Mnx (*n* = 4 knees)	Partial lateral meniscectomy/lateral hemidiscectomy; approximately 50% of the lateral meniscus was removed.	No intra‐articular HA, HA + P6, saline, or vehicle injection.	Meniscectomy‐only injury reference group.
Mnx + HA (*n* = 6 knees)	Partial lateral meniscectomy/lateral hemidiscectomy; approximately 50% of the lateral meniscus was removed.	2 mL BDDE‐cross‐linked HA at weeks 0, 1, 2, and 4.	HA carrier comparator after meniscectomy.
Mnx + HA + P6 (*n* = 6 knees)	Partial lateral meniscectomy/lateral hemidiscectomy; approximately 50% of the lateral meniscus was removed.	2 mL of the same HA carrier supplemented with 50 μg/mL P6 at weeks 0, 1, 2, and 4.	Primary test formulation after meniscectomy.

The treatment‐level operated cohort comprised 16 surgically injured knees from 8 sheep: Mnx (*n* = 4 knees), Mnx + HA (*n* = 6 knees), and Mnx + HA + P6 (*n* = 6 knees). In addition, 7 Intact no‐surgery/no‐injection knees were included as baseline reference tissue. The study was designed to determine whether P6 supplementation conferred additional repair‐associated benefit beyond that of the cross‐linked HA carrier. The primary biological comparison was therefore Mnx + HA + P6 versus Mnx + HA. Mnx + HA versus Mnx and Mnx + HA + P6 versus Mnx were treated as injury‐reference comparisons, while comparisons with Intact were used to contextualize meniscectomy‐associated tissue changes relative to no‐surgery/no‐injection reference tissue.

### 2.2 Materials and HA + P6 Hydrogel Preparation

The HA carrier was prepared from pharmaceutical‐grade high‐molecular‐weight sodium hyaluronate using BDDE crosslinking, as described previously [39]. Briefly, sodium hyaluronate was dissolved at 10 w/v% during the manufacturing/crosslinking step, cross‐linked with BDDE, dialyzed, granulated, and adjusted to a final injectable HA concentration of 20 mg/mL before syringe filling. The HA + P6 formulation contained the same BDDE‐cross‐linked HA carrier supplemented with 50 μg/mL P6. The peptide concentration was selected based on prior peptide bioactivity and HA + P6 studies [33, 34, 35, 38].

Regenarti AS manufactured the HA and HA + P6 preparations in accordance with ISO 13485:2016‐compliant procedures. The hydrogels were prefilled into syringes, sterilized by autoclaving, and injected using sterile 21‐gauge needles. Release testing was performed according to predefined quality‐control criteria, including endotoxin ≤ 0.5 EU/g, bioburden ≤ 100 CFU/mL, and residual BDDE ≤ 2 mg/kg.

The hydrogels used here were based on a previously characterized BDDE‐cross‐linked HA platform [39, 40, 41, 42, 43]. Post‐sterilization rheology, enzymatic degradation, peptide release, peptide integrity after autoclaving, residual BDDE, endotoxin, and sterility release criteria have been reported previously [40, 41, 42, 43]. The present in vivo study evaluates tissue responses to the manufactured HA and HA + P6 formulations. It does not make new independent claims regarding intra‐articular residence time, peptide release, peptide retention, or network reinforcement beyond what is supported by the biological outcomes reported here.

### 2.3 Animals, Ethics, Allocation, and Blinding

Eight adult female sheep were included. Facility records identified the animals as ovine females transported to the Rof Codina Foundation/Cebiovet animal facility in Lugo, Spain, before surgery. At surgery, the animals were approximately 5.0–5.5 years old. Body weight was recorded for anesthetic dosing, and the animals were housed under veterinary supervision with access to water and standard ovine feed.

All procedures were approved by the Ethical Committee of the Rof Codina Foundation and the regional government of Xunta de Galicia (approval number 03/18/LU‐001) and complied with applicable animal‐welfare regulations. The study was a controlled preclinical pilot study constrained by animal availability and ethical and logistical considerations; no prospective sample size calculation was available for the structural endpoint.

In the operated cohort, each sheep underwent bilateral stifle surgery, with both knees contributing to the study. Within each stifle, the procedure was restricted to the lateral compartment. Injured knees were allocated to Mnx, Mnx + HA, or Mnx + HA + P6 using a predesigned allocation form; the left/right side was not used as the treatment‐defining variable. Mnx knees received meniscectomy only and no intra‐articular injection. Mnx + HA and Mnx + HA + P6 knees received the assigned intra‐articular formulation. Surgeons were therefore not blinded to treatment allocation. Investigators performing microCT reconstruction, histological scoring, image quantification, and statistical analysis were blinded to group allocation wherever feasible.

### 2.4 Surgery, Injections, and Post‐Operative Monitoring

Meniscal injury‐associated OA‐like change was induced by bilateral partial lateral meniscectomy/lateral hemidiscectomy, comparable to established ovine meniscal injury and meniscectomy models [14, 15, 44]. Animals were placed under general anesthesia according to institutional veterinary protocols for premedication, induction, maintenance, perioperative analgesia, and antibiotic prophylaxis. Through a lateral parapatellar arthrotomy, the lateral meniscus was exposed. Approximately 50% of the lateral meniscus was freed by incision and removed. The remaining meniscal rim and resection site were left unrepaired. Cruciate and collateral ligaments were protected and left intact. The joint was irrigated, hemostasis was confirmed, and the wound was closed in three layers: capsule, muscle, and skin.

Mnx + HA and Mnx + HA + P6 knees received 2 mL of the assigned formulation intra‐articularly immediately post‐operatively at week 0 and again at weeks 1, 2, and 4, using a sterile 21‐gauge needle via a standard parapatellar approach under aseptic conditions. Mnx knees underwent the same partial lateral meniscectomy but received no intra‐articular injection. Intact knees underwent no meniscectomy and no injection. Repeat injections were performed under restraint and light sedation as required to minimize stress and support accurate intra‐articular delivery.

No post‐operative activity restriction was applied. Operated animals were monitored until recovery from anesthesia and then examined regularly by veterinary personnel. Monitoring included wound status, joint swelling, gait or lameness, body weight, appetite, general health, adverse events, unscheduled veterinary interventions, deviations from the injection schedule, and exclusions. At 12 weeks post‐surgery, operated animals were euthanized under veterinary supervision using an approved overdose protocol. Knees were harvested en bloc and fixed in 4% paraformaldehyde for histological analysis [45, 46]. Intact reference knees were processed using the same available endpoint workflows for which the corresponding data were available.

### 2.5 Micro‐Computed Tomography

Periarticular specimens were scanned ex vivo using a SkyScan 2211 system (Bruker microCT, Kontich, Belgium) operated at 120 kV and 50 μA with a 0.5 mm copper filter and a nominal isotropic voxel size of 30 μm. Projection data were acquired with frame averaging, reconstructed in NRecon 1.7.5.9, reoriented in DataViewer 1.7.0, and analyzed in CTAn 1.23.0.2. Identical reconstruction and analysis settings were applied across specimens.

A standardized periarticular osteochondral volume of interest was applied to all specimens. Before segmentation, images were filtered using three‐dimensional anisotropic diffusion filtering with the Perona‐Malik high‐contrast edge‐preserving algorithm. Bone was segmented using automatic Otsu thresholding in three‐dimensional space with a dark background; the lower and upper gray‐value thresholds were 88 and 255, respectively. Trabecular and cortical/subchondral masks were generated using the same predefined batch workflow and analyzed separately.

Trabecular morphometric outcomes included tissue volume, bone volume, percent bone volume/BV/TV, bone surface, bone surface‐to‐volume ratio, bone surface density, trabecular thickness, trabecular separation, trabecular number, trabecular pattern factor, structure model index, fractal dimension, degree of anisotropy, and thickness/separation distributions. Cortical/subchondral outcomes included tissue volume, bone volume, percent bone volume, bone surface, bone surface‐to‐volume ratio, bone surface density, local thickness, fractal dimension, and degree of anisotropy. Morphometric outputs were exported in CTAn units: lengths in μm, surfaces in μm^2^, volumes in μm^3^, inverse‐length variables in μm^−1^, and BV/TV as a percentage.

### 2.6 Histology, Immunohistochemistry, and Image Analysis

After microCT, specimens were dehydrated, embedded in methyl methacrylate, and polymerized at −20°C [47]. Embedded osteochondral specimens were sectioned through the central operated region. Sections were cut at 5 μm onto Kawamoto film using a motorized Thermo/Microm HM 355S microtome [41]. Serial sections were collected to assess tissue morphology, matrix composition, collagen organization, proteoglycan/glycosaminoglycan‐rich matrix, and immunohistochemical marker expression.

Hematoxylin and eosin (H&E) staining assessed general tissue architecture, cellularity, cartilage morphology, surface integrity, subchondral bone morphology, trabecular appearance, and continuity of osteochondral structures [42]. Movat pentachrome staining assessed matrix composition and collagen‐rich tissue within predefined regions of interest [43]. Picrosirius Red assessed collagen‐rich matrix organization. Alcian Blue and Safranin O assessed glycosaminoglycan‐ and proteoglycan‐rich matrix within cartilage and cartilage‐like tissue [48]. H&E‐based scoring used predefined ordinal endpoints aligned, where applicable, with OARSI sheep/goat histopathology recommendations [44].

Type II collagen (COL2) and MMP13 immunohistochemistry assessed cartilage matrix and catabolic matrix remodeling [49]. Sections were blocked with Bloxall blocking solution, incubated with primary antibody diluted in DAKO‐Diluent, incubated with Vectastain ABC alkaline phosphatase secondary detection reagent, and visualized with Vector Red alkaline phosphatase substrate [45, 50]. Type II collagen was detected with rabbit polyclonal anti‐COL2 (Abcam ab34712; 1:400), and MMP13 was detected with rabbit monoclonal anti‐MMP13 (Abcam ab21962; 1:500).

Slides were digitized on an AxioScan Z1 slide scanner and analyzed in ImageJ 1.53f51 [36]. Images were acquired using standardized magnification, illumination, exposure, white balance, and region‐of‐interest criteria for each staining protocol. Cartilage, osteochondral interface, subchondral plate, and trabecular bone regions were analyzed separately, where possible, because the stains did not have uniform biological meaning across compartments.

### 2.7 Statistical Analysis

The animal was treated as the highest‐level clustering unit. The knee/sample was treated as the treatment‐level observational unit only after accounting for sheep identity when valid identifiers were available. Repeated sections, images, fields, regions of interest, and exported software rows were aggregated into animal/knee/sample‐level endpoint values prior to inferential analysis and were not considered independent biological replicates. Endpoint‐specific animal and knee/sample coverage was audited before modeling because microCT, H&E histology, and IHC/histochemical datasets had different endpoint availability. Identifier coverage, endpoint modelability, exclusions, and group‐specific animal/knee counts are summarized in Supporting Information S1: Tables S1–S3 and Supporting Information S1: Figures S7 and S8; sensitivity analyses are summarized in Supporting Information S1: Table S8.

Graphs were plotted in GraphPad Prism 10 and R. All analyses were performed using a reproducible *R* 4.6.0 workflow for statistical modeling and sensitivity analyses. Treatment labels were standardized as Intact, Mnx, Mnx + HA, and Mnx + HA + P6. Intact denotes no‐surgery/no‐injection reference knees; Mnx denotes partial lateral meniscectomy‐only knees; Mnx + HA denotes meniscectomy plus crosslinked HA; and Mnx + HA + P6 denotes meniscectomy plus the same HA carrier supplemented with P6. The primary biological contrast was Mnx + HA + P6 versus Mnx + HA, testing whether P6 provided added benefit beyond the HA carrier. Mnx + HA versus Mnx and Mnx + HA + P6 versus Mnx were injury‐reference comparisons, while comparisons with Intact were used to contextualize meniscectomy‐associated changes relative to no‐surgery/no‐injection reference tissue.

For continuous endpoints, group differences were estimated using linear mixed models with treatment group as a fixed effect and sheep identity as a random intercept when the random effect was identifiable. When the random intercept was not identifiable because each animal contributed only one modelable observation for a given endpoint, an animal‐ or knee‐level fixed‐effects linear model was used and explicitly flagged. Estimated marginal means and prespecified contrasts were obtained using *emmeans*. Corrected contrasts are reported as mean differences with 95% confidence intervals, *p* values, and Benjamini–Hochberg false‐discovery‐rate‐adjusted *q* values. Multiplicity was controlled within endpoint families and globally across the broader set of exploratory models.

Trabecular microCT endpoints were treated as the principal structural readouts, with percent trabecular bone volume/BV/TV as the primary structural endpoint and trabecular separation, trabecular thickness, and trabecular number as key secondary structural endpoints. Cortical/subchondral and surface microCT outcomes were analyzed as related exploratory structural readouts. H&E‐derived histological scores were treated as supportive semi‐quantitative tissue‐level outcomes. Because these scores are ordinal, ordinal cumulative‐link models were evaluated as sensitivity analyses where endpoint structure and group coverage were allowed. When ordinal models were not identifiable or were unstable, the corresponding animal/knee‐level mixed‐model or fixed‐effect summaries were retained and interpreted cautiously.

IHC and histochemical measurements were treated as exploratory tissue‐pattern readouts. The sample‐level IHC/histochemical mean dataset was analyzed separately from the broader animal/knee‐level IHC/histomorphometry mixed‐model screen. Bounded IHC/histochemical percentage endpoints were additionally assessed using beta or fractional regression sensitivity models, where boundary values and model identifiability were allowed. Light/dark color‐fraction variables were interpreted as image‐derived descriptors rather than independently validated biological endpoints. Evidence from IHC/histochemical analyses was therefore considered supportive and exploratory unless consistent across model type, contrast direction, and multiplicity correction.

Robustness analyses included endpoint‐specific *n* audits, model diagnostics, heteroscedastic generalized least‐squares sensitivity models where residual variance patterns suggested group‐specific dispersion, animal‐cluster bootstrap confidence intervals for key endpoints, animal‐level permutation tests where sample size permitted, and leave‐one‐animal‐out influence checks. A normal‐approximation Bayesian sensitivity analysis was performed for key microCT endpoints to quantify uncertainty and posterior support for favorable treatment effects. An exploratory direction‐aligned repair score was calculated only as a secondary integrative summary and was not used to replace endpoint‐level inference; the corresponding exploratory repair‐score contrasts are summarized in Supporting Information S1: Table S9. Precision and sample‐size simulations were performed to inform future confirmatory study design and were not used to claim the adequacy of the present pilot. The sensitivity‐analysis overview is provided in Supporting Information S1: Table S8, compact primary‐contrast robustness outputs in Supporting Information S1: Table S12, and future power/precision simulations in Supporting Information S1: Figure S10 and Supporting Information S1: Table S10.

Candidate outliers were flagged using conservative statistical and graphical rules, but were not removed unless a technical artifact, duplicate export, unresolved identifier, or measurement error was documented. For reproducibility, cleaned endpoint name, endpoint family, treatment group, sheep identifier, sample/knee identifier, section or image identifier where applicable, aggregation status, inclusion status, exclusion reason, model type, and curation status were retained in the analysis datasets and audit tables.

## 3 Results

### 3.1 Experimental Workflow and Outcome Hierarchy

The operated cohort comprised eight sheep that underwent bilateral partial lateral meniscectomy/lateral hemidiscectomy, yielding 16 surgically injured knees allocated to Mnx, Mnx + HA, or Mnx + HA + P6. Mnx + HA and Mnx + HA + P6 knees received repeated intra‐articular injections at weeks 0, 1, 2, and 4, whereas Mnx knees underwent the same meniscectomy procedure but received no intra‐articular injection. Intact no‐surgery/no‐injection knees were included as baseline reference tissue. Operated animals were euthanized at 12 weeks for microCT, histology, immunohistochemistry, and histochemical image analysis.

**FIGURE 1:**
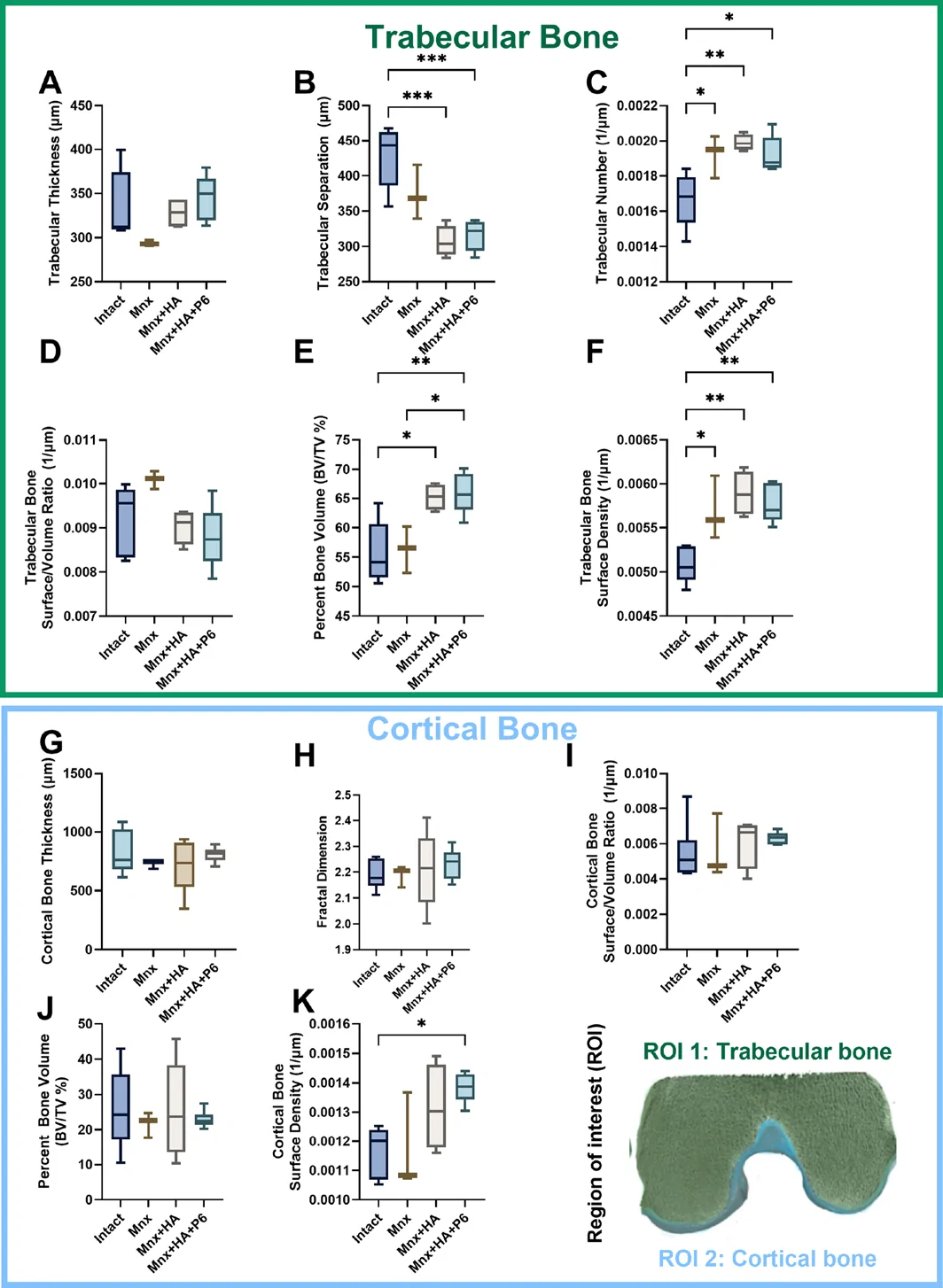
MicroCT analysis of trabecular and cortical/subchondral bone architecture after partial lateral meniscectomy and intra‐articular treatment. Panels (A–F) show trabecular morphometric parameters including trabecular thickness (A), trabecular separation (B), trabecular number (C), trabecular bone surface‐to‐volume ratio (D), trabecular bone volume fraction (BV/TV) (E), and trabecular bone surface density (F). Panels (G–K) show cortical/subchondral morphometric parameters including cortical thickness (G), cortical bone volume fraction (H), cortical bone surface‐to‐volume ratio (I), cortical fractal dimension (J), and cortical bone surface density (K). Box‐and‐whisker plots show the distribution of measurements in the Intact, Mnx, Mnx+HA, and Mnx+HA+P6 groups.

The outcome hierarchy prioritized corrected animal/knee‐level inference. Trabecular microCT morphometry was retained as the principal structural readout, with percent trabecular bone volume/BV/TV as the primary structural endpoint and trabecular separation, trabecular thickness, and trabecular number as key secondary structural endpoints. The exploratory morphometry distributions in Figure 1 were used to visualize group‐level structural patterns, whereas corrected animal/knee‐level contrasts were used for inferential interpretation. Semi‐quantitative histological scoring and image‐based quantification of matrix and catabolic markers were used as supportive tissue‐level readouts. The primary biological comparison was between Mnx + HA + P6 and Mnx + HA to test whether P6 provided an added benefit beyond the HA carrier.

### 3.2 Trabecular Architecture Showed the Clearest MicroCT Response

**FIGURE 2:**
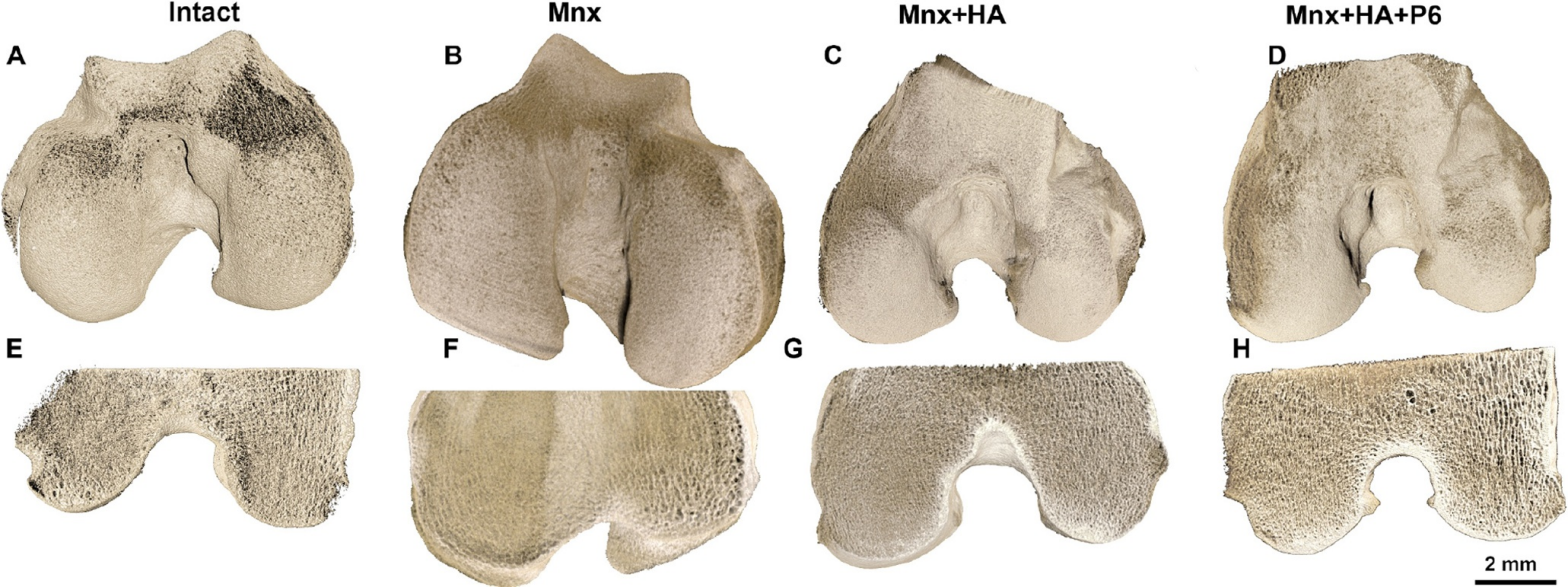
Representative 3D microCT reconstructions, virtual cross‐sections, and region‐of‐interest definition for osteochondral morphometry. Representative micro‐computed tomography images of ovine knee osteochondral specimens from the Intact (A, E), Mnx (B, F), Mnx + HA (C, G), and Mnx + HA + P6 (D, H) groups. Intact denotes no‐surgery/no‐injection reference knees; Mnx denotes partial lateral meniscectomy/lateral hemidiscectomy without intra‐articular injection; Mnx + HA denotes meniscectomy followed by cross‐linked HA injection; and Mnx + HA + P6 denotes meniscectomy followed by injection of the same HA carrier supplemented with P6. The figure provides anatomical context for compartment‐specific microCT analysis and the operated osteochondral region.

Representative 3D microCT reconstructions, virtual cross‐sections, and region‐of‐interest definitions are shown in Figure 2. Exploratory microCT morphometry showed the clearest group‐level separation in the trabecular compartment (Figure 1A–F). The figure summarizes trabecular thickness, trabecular separation, trabecular number, trabecular bone surface‐to‐volume ratio, percent trabecular bone volume/BV/TV, and trabecular bone surface density across Intact, Mnx, Mnx + HA, and Mnx + HA + P6 knees.

**FIGURE 3:**
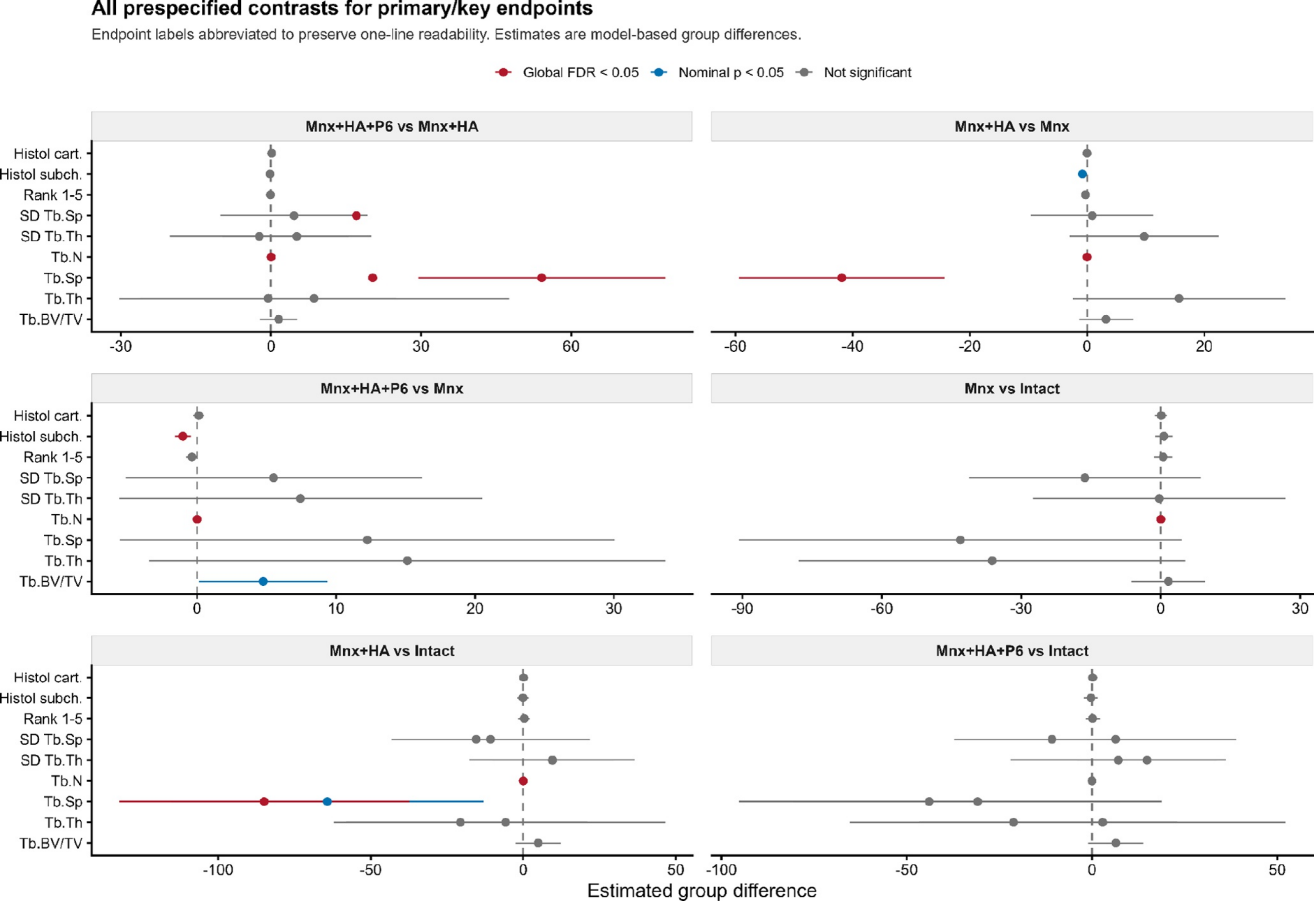
Prespecified model‐based contrasts for primary and key secondary endpoints. Forest plot of corrected animal/knee‐level model estimates for prespecified contrasts across primary and key secondary structural and histological endpoints. Points indicate estimated group differences, and horizontal bars indicate 95% confidence intervals. The dashed vertical line denotes no estimated group difference. The separate plot sections are labeled by contrast: Mnx + HA + P6 vs Mnx + HA, Mnx + HA vs Mnx, Mnx + HA + P6 vs Mnx, Mnx vs Intact, Mnx + HA vs Intact, and Mnx + HA + P6 vs Intact. Red points indicate contrasts supported after global false‐discovery‐rate correction (global FDR < 0.05), blue points indicate nominal *p* < 0.05 without global FDR support, and gray points indicate non‐significant contrasts. Endpoint labels were abbreviated for readability: Histol cart., histological cartilage summary score; Histol subch., histological subchondral summary score; Rank 1–5, global histological rank; SD Tb.Sp, standard deviation of trabecular separation; SD Tb.Th, standard deviation of trabecular thickness; Tb.N, trabecular number; Tb.Sp, trabecular separation; Tb.Th, trabecular thickness; Tb.BV/TV, percent trabecular bone volume. Statistical significance indicates evidence for a group difference, but the biological direction of benefit depends on the endpoint; the primary interpretation remains whether Mnx + HA + P6 provides added benefit beyond Mnx + HA.

The most consistent visual pattern was observed for trabecular BV/TV and trabecular separation. Mnx + HA and Mnx + HA + P6 showed higher trabecular BV/TV than Mnx, whereas trabecular separation was lower in the HA‐treated groups than in Mnx. Trabecular number and trabecular bone surface density also showed exploratory group separation, with a generally more favorable trabecular architecture in the HA‐treated groups. The asterisk annotations in Figure 2 indicate exploratory pairwise differences, but these plots should not be interpreted as proof of peptide‐specific efficacy because they do not by themselves establish the primary Mnx + HA + P6 versus Mnx + HA contrast after endpoint‐specific animal/knee‐level correction. Formal corrected contrasts are presented below in Figure 3 and Table 2.

**TABLE 2:** Corrected structural contrasts and interpretation.

Endpoint/contrast	Corrected animal/knee‐level result	Multiplicity and sensitivity context	Interpretation
Percent trabecular bone volume/BV/TV, Mnx + HA + P6 versus Mnx + HA	Mean difference +1.52% points (95% CI −2.22 to 5.26); *p* = 0.427.	Family *q* = 0.762; global *q* = 0.909; 10 animals/17 knees.	Primary structural contrast does not support a robust added P6 benefit beyond HA.
Percent trabecular bone volume/BV/TV, Mnx + HA versus Mnx	Mean difference +3.23% points (95% CI −1.39 to 7.85); *p* = 0.170.	Not FDR‐supported.	Directionally favorable HA‐associated pattern versus meniscectomy‐only injury, but not statistically robust.
Percent trabecular bone volume/BV/TV, Mnx + HA + P6 versus Mnx	Mean difference +4.75% points (95% CI 0.13–9.37); *p* = 0.044.	Nominal only; family *q* = 0.298; global *q* = 0.515.	Exploratory treatment‐versus‐injury signal; not sufficient to claim corrected efficacy.
Key secondary trabecular endpoints, Mnx + HA + P6 versus Mnx + HA	Endpoint‐specific estimates varied and did not show a consistently favorable corrected effect of P6 supplementation.	Full endpoint‐specific results and sensitivity analyses are provided in the supporting information.	Secondary trabecular endpoints do not overturn the primary conclusion.
Cortical/subchondral microCT endpoints, Mnx + HA + P6 versus Mnx + HA	No consistent corrected evidence of peptide‐specific benefit was identified.	Interpreted as an exploratory structural context.	Cortical/subchondral outcomes should not be used to claim Mnx + HA + P6 superiority.

### 3.3 Cortical/Subchondral Outcomes Showed a Narrower Response Pattern

Cortical/subchondral outcomes showed less consistent separation than trabecular outcomes (Figure 1G–K). Cortical thickness, cortical fractal dimension, cortical bone surface‐to‐volume ratio, and percent cortical bone volume did not show a broad or coherent treatment‐associated pattern across the groups. Cortical bone surface density showed the clearest exploratory cortical/subchondral signal, with an annotated group difference in Figure 1K, but this should be interpreted in the context of structural relationships rather than as evidence of a peptide‐specific effect. Overall, cortical/subchondral outcomes were less coherent than trabecular outcomes and were retained as exploratory structural readouts.

### 3.4 Representative Histology and Semi‐Quantitative Scoring

**FIGURE 4:**
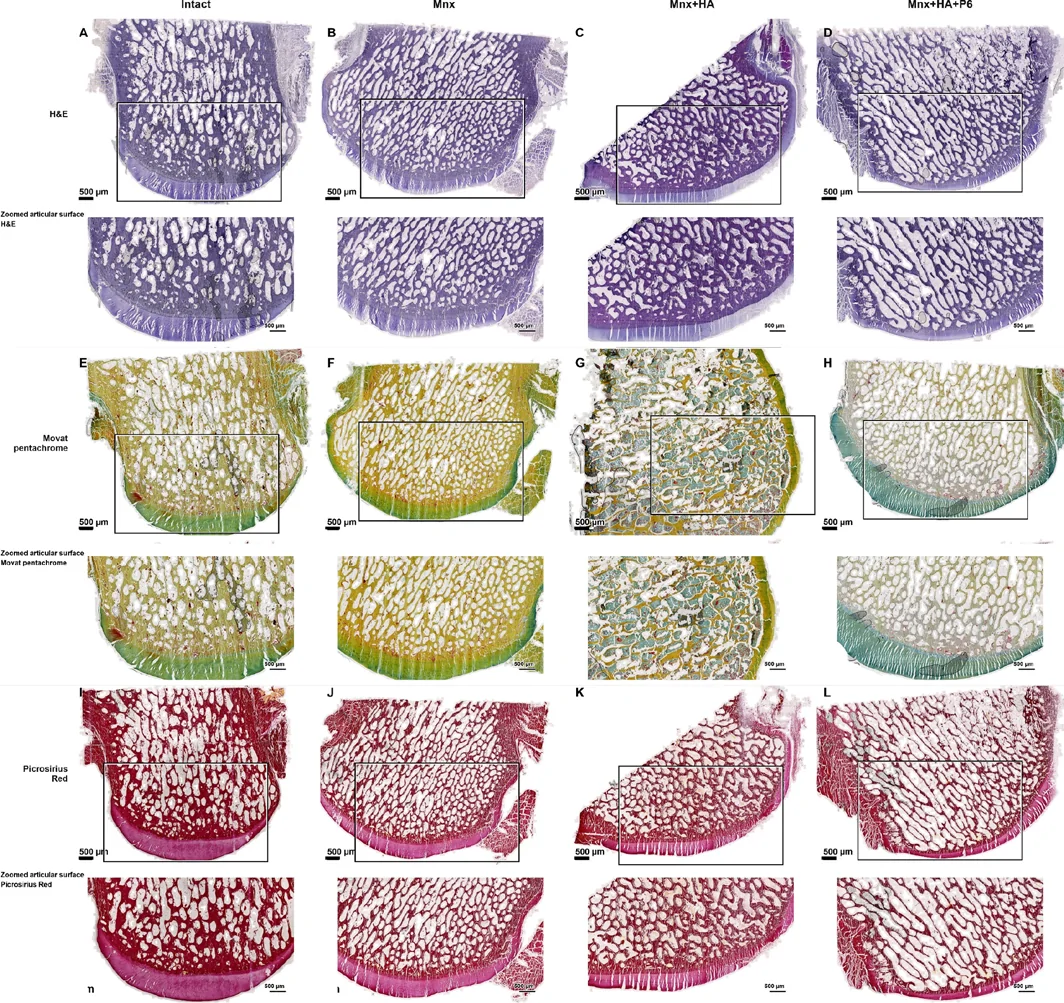
Representative osteochondral histology after partial lateral meniscectomy and intra‐articular treatment. Representative low‐magnification osteochondral sections from Intact (A, E, I), Mnx (B, F, J), Mnx + HA (C, G, K), and Mnx + HA + P6 (D, H, L) knees are shown for H&E, Movat pentachrome, and Picrosirius Red staining. Intact denotes no‐surgery/no‐injection reference knees; Mnx denotes partial lateral meniscectomy/lateral hemidiscectomy without intra‐articular injection; Mnx + HA denotes meniscectomy followed by cross‐linked HA injection; and Mnx + HA + P6 denotes meniscectomy followed by injection of the same HA carrier supplemented with P6. The images illustrate the overall osteochondral architecture and the distribution of the collagen‐rich matrix. Representative fields provide anatomical and tissue‐level context but were not treated as independent evidence of treatment efficacy.

Representative H&E, Movat pentachrome, and Picrosirius Red‐stained osteochondral sections are shown in Figure 4 for the Intact, Mnx, Mnx + HA, and Mnx + HA + P6 groups. These sections illustrate the relationships among the articular surface, cartilage‐associated matrix, subchondral bone plate, and underlying trabecular bone. H&E was used to assess general tissue architecture, cellular distribution, surface integrity, cartilage defects, and subchondral/trabecular organization. Movat pentachrome provided a broader matrix readout, and Picrosirius Red visualized collagen‐rich matrix. Additional orientation‐specific histological panels and articular‐surface close‐ups are provided in Supporting Information S1: Figures S1 and S5.

Across representative sections, all groups showed identifiable osteochondral architecture with a cartilage‐associated peripheral zone and underlying trabecular network. The Mnx fields showed a more injury‐associated architectural disturbance in selected regions, whereas Mnx + HA and Mnx + HA + P6 fields often showed a more compact trabecular/subchondral appearance. These visual patterns are consistent with the microCT findings, but representative morphology alone was not treated as evidence of treatment efficacy.

**FIGURE 5:**
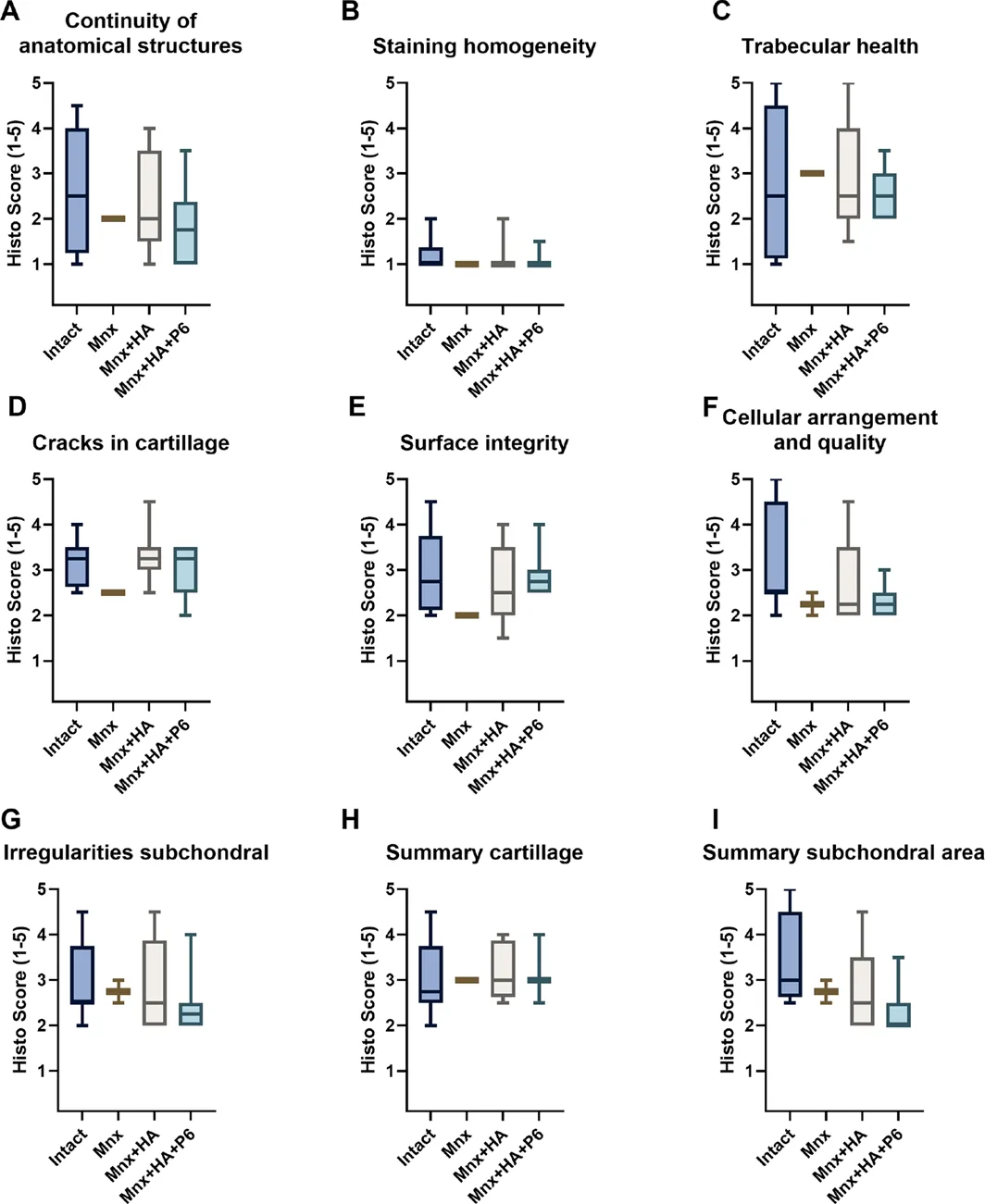
Semi‐quantitative histological evaluation of H&E‐stained osteochondral sections. Panels show continuity of anatomical structures (A), staining homogeneity (B), trabecular health (C), cartilage cracks (D), surface integrity (E), cellular arrangement and quality (F), subchondral irregularities (G), summary cartilage score (H), and summary subchondral score (I). Box‐and‐whisker plots summarize the distributions for the Intact, Mnx, Mnx + HA, and Mnx + HA + P6 groups.

Semi‐quantitative H&E‐based scoring provided supportive tissue‐level information rather than independent confirmatory evidence (Figure 5). Histological summary scores in Figure 3 support the same cautious interpretation: selected treatment‐versus‐Mnx tissue patterns were observed, but the direct Mnx + HA + P6 versus Mnx + HA contrast was not significant.

The subchondral summary score showed a more favorable pattern in the treated groups relative to Mnx, with lower scores indicating less abnormality. Mnx + HA + P6 showed a lower subchondral summary score than Mnx (estimate −1.02; 95% CI −1.61 to −0.44; *p* < 0.001; family *q* = 0.045; global *q* = 0.017). Mnx + HA also showed a nominally lower score than Mnx (estimate −0.81; 95% CI −1.40 to −0.23; *p* = 0.007), although this was not supported after the same correction hierarchy. The direct Mnx + HA + P6 versus Mnx + HA contrast was not significant (estimate −0.21; 95% CI −0.70 to 0.27; *p* = 0.391). Individual H&E features, including cellular arrangement, anatomical continuity, cartilage cracking, surface integrity, trabecular health, and subchondral irregularity, did not show a consistent pattern establishing the additive benefit of P6 over HA.

Taken together, histological scoring supports the broader interpretation that HA‐based treatment was associated with selected tissue‐level patterns after meniscectomy, particularly in the subchondral region. However, the histological data do not establish consistent superiority of Mnx + HA + P6 over Mnx + HA and should be interpreted as a supportive context for the microCT findings.

### 3.5 Matrix and Catabolic‐Marker Staining

**FIGURE 6:**
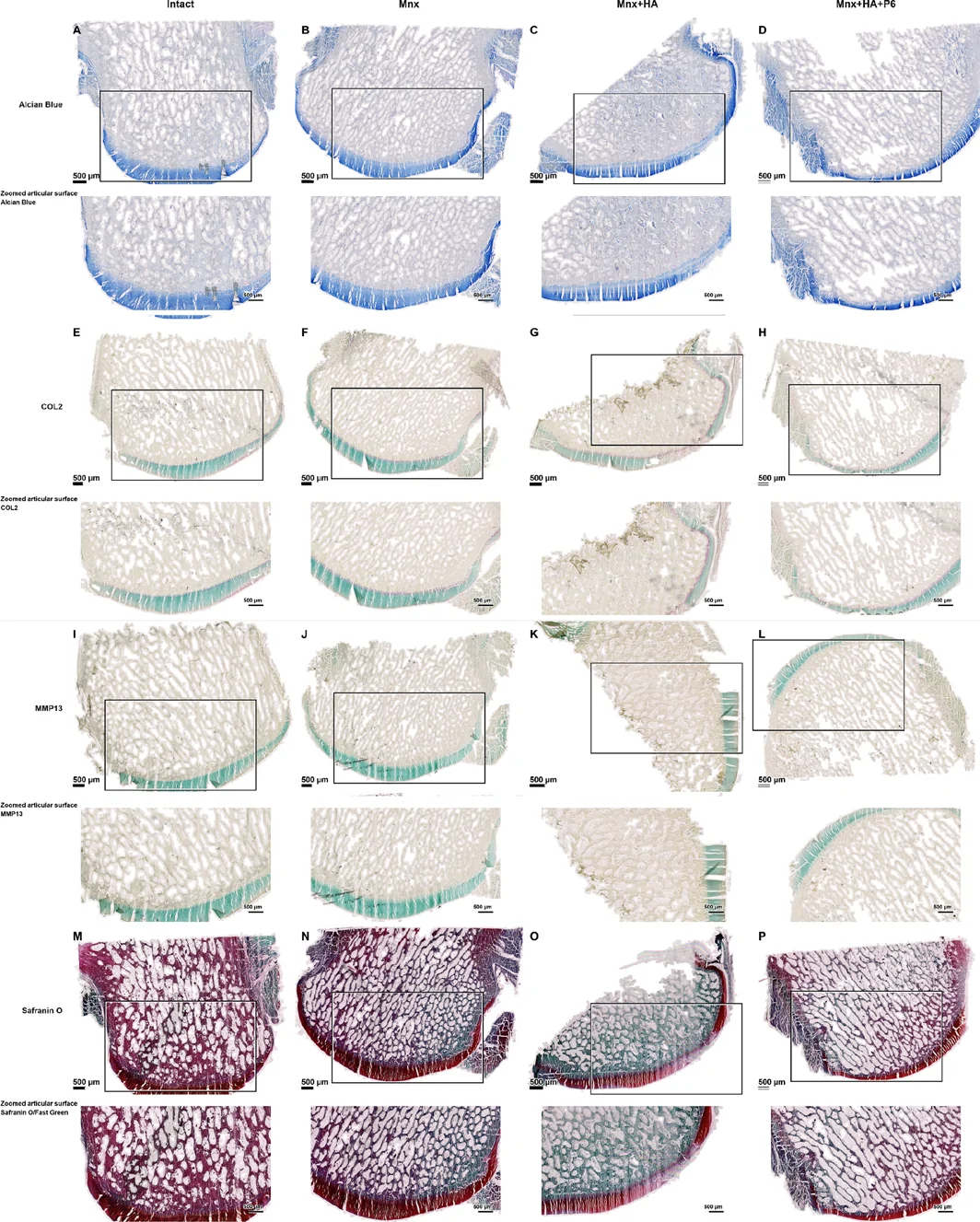
Representative histochemical and immunohistochemical staining of osteochondral tissue from the Intact, Mnx, Mnx + HA, and Mnx + HA + P6 groups. Alcian Blue staining is shown in panels (A–D), type II collagen (COL2) immunohistochemistry in panels (E–H), MMP13 immunohistochemistry in panels (I–L), and Safranin O staining in panels (M–P). Within each staining modality, the upper panels show representative low‐magnification sections and the corresponding lower panels show higher‐magnification views of the articular surface region. Scale bars = 500 μm.

Representative sections of Alcian Blue, type II collagen, MMP13, and Safranin O are shown in Figure 6 for the Intact, Mnx, Mnx + HA, and Mnx + HA + P6 groups. Additional orientation‐specific histochemical/IHC panels and collagen‐associated articular‐surface close‐ups are provided in Supporting Information S1: Figures S2–S4 and S6. Alcian Blue and Safranin O were used as proteoglycan/glycosaminoglycan‐associated matrix readouts, with the strongest staining expected in cartilage‐associated matrix rather than mineralized trabecular bone. Type II collagen was used as a hyaline cartilage matrix‐associated marker, whereas MMP13 was used as a catabolic matrix‐remodeling marker.

Across the representative sections, Alcian Blue and Safranin O staining were concentrated along the cartilage‐rich surface and peripheral tissue regions. The subchondral and trabecular compartments showed weaker and more heterogeneous staining. Type II collagen staining was primarily surface‐ and cartilage‐associated, without a uniform increase across orientations or treatment groups. MMP13 staining was focal and regionally variable. These findings support compartment‐specific analysis rather than pooling cartilage and subchondral tissue into a single readout.

**FIGURE 7:**
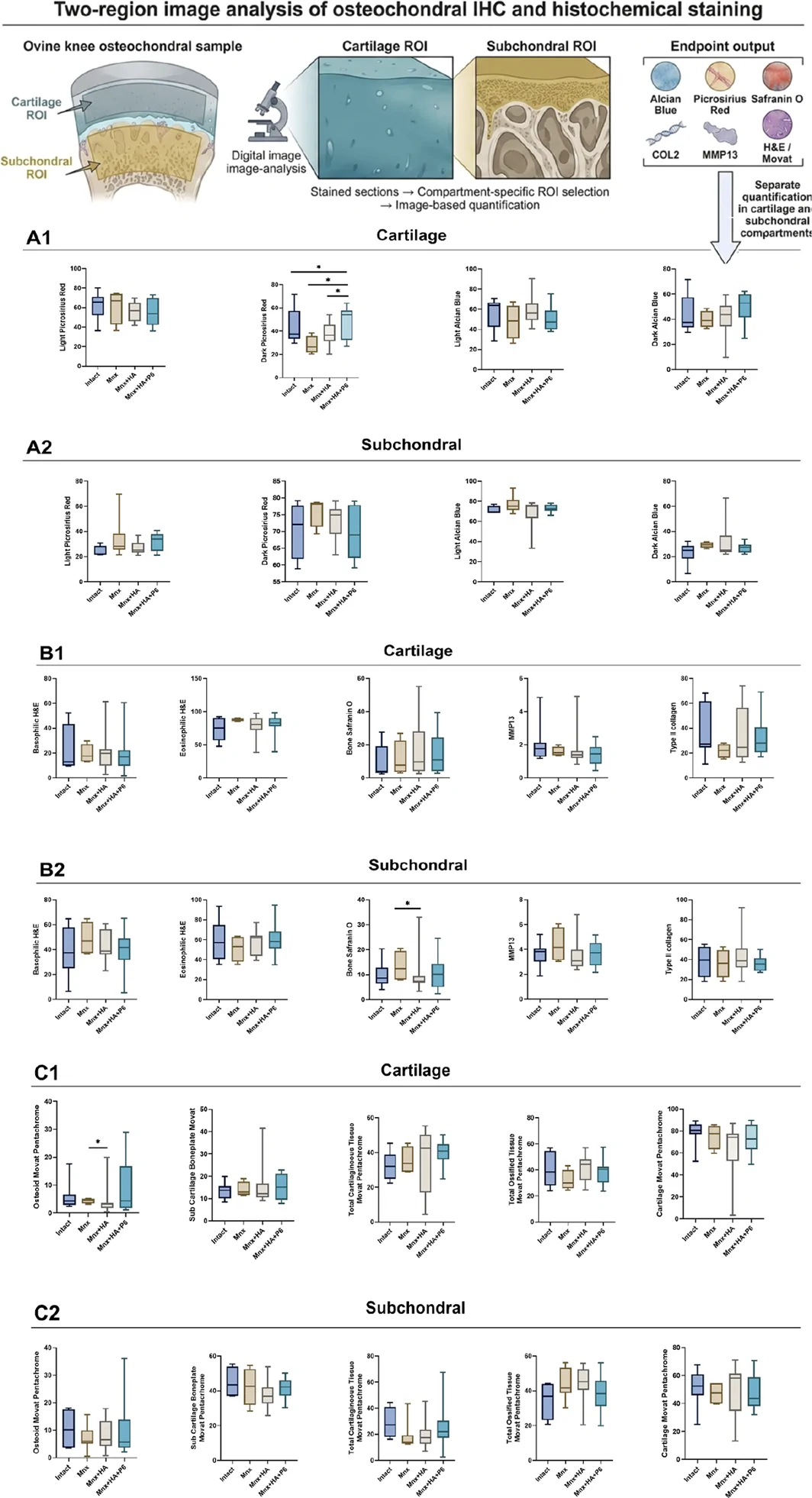
The upper schematic illustrates the two‐region image‐analysis workflow. Cartilage and subchondral regions of interest were selected from stained osteochondral sections and quantified separately. Panel A1 shows cartilage‐region outcomes from the first endpoint block, and panel A2 shows the corresponding subchondral‐region outcomes. Panel B1 shows cartilage‐region outcomes from the second endpoint block, and panel B2 shows the corresponding subchondral‐region outcomes. Panel C1 shows cartilage‐region outcomes from the third endpoint block, and panel C2 shows the corresponding subchondral‐region outcomes. Endpoint names are shown above the individual plots within each panel block. Data are shown for the Intact, Mnx, Mnx+HA, and Mnx+HA+P6 groups. Box‐and‐whisker plots summarize the distributions within each group. Asterisks indicate exploratory pairwise differences shown in the figure; these image‐derived outcomes were interpreted as exploratory tissue‐pattern readouts rather than confirmatory evidence of peptide‐specific efficacy.

Image‐based quantification was therefore performed separately in the cartilage and subchondral regions (Figure 7). The strongest cartilage‐region signal was observed for Dark Picrosirius Red. In the broader animal/knee‐level IHC/histomorphometry mixed‐model analysis, cartilage Dark Picrosirius Red was higher in Mnx + HA + P6 than in Mnx + HA by 11.69% points (95% CI 5.32 to 18.06; *p* = 0.00032; global *q* = 0.0106; family *q* = 0.0537; Supporting Information S1: Table S7; Supporting Information S1: Figure S9). This signal is therefore globally FDR‐supported but narrowly non‐significant after IHC‐family FDR correction, and it is interpreted as an exploratory collagen‐associated matrix‐pattern signal rather than confirmatory evidence of peptide‐specific superiority. Additional pairwise annotations in Figure 7 also indicate higher cartilage Dark Picrosirius Red in Mnx + HA + P6 relative to selected reference groups, but these comparisons are interpreted with caution in the broader multiplicity context.

Other image‐derived signals were more limited. Figure 7 shows selected nominal pairwise differences for subchondral Bone Safranin O and cartilage Osteoid Movat pentachrome. In the mixed‐model summary, cartilage Bone Safranin O was higher in Mnx + HA than in Mnx (estimate 9.43% points; 95% CI 1.38 to 17.47; *p* = 0.022), and cartilage Movat showed a nominal Mnx + HA versus Mnx contrast (estimate −14.98% points; 95% CI −29.40 to −0.55; *p* = 0.042). These findings did not provide a consistent pattern of Mnx + HA + P6 superiority over Mnx + HA and should be considered exploratory.

Most other cartilage and subchondral markers, including COL2, MMP13, Alcian Blue‐derived fractions, H&E‐derived fractions, and most Movat‐derived fractions, did not show robust group separation. The updated sample‐level IHC/histochemical mean results likewise did not show FDR‐robust group‐level separation and did not identify a nominally significant direct Mnx + HA + P6 versus Mnx + HA contrast. Taken together, the staining data indicate selected matrix‐associated tissue‐pattern signals, particularly involving cartilage Dark Picrosirius Red, but do not establish the molecular superiority of Mnx + HA + P6 over Mnx + HA.

**FIGURE 8:**
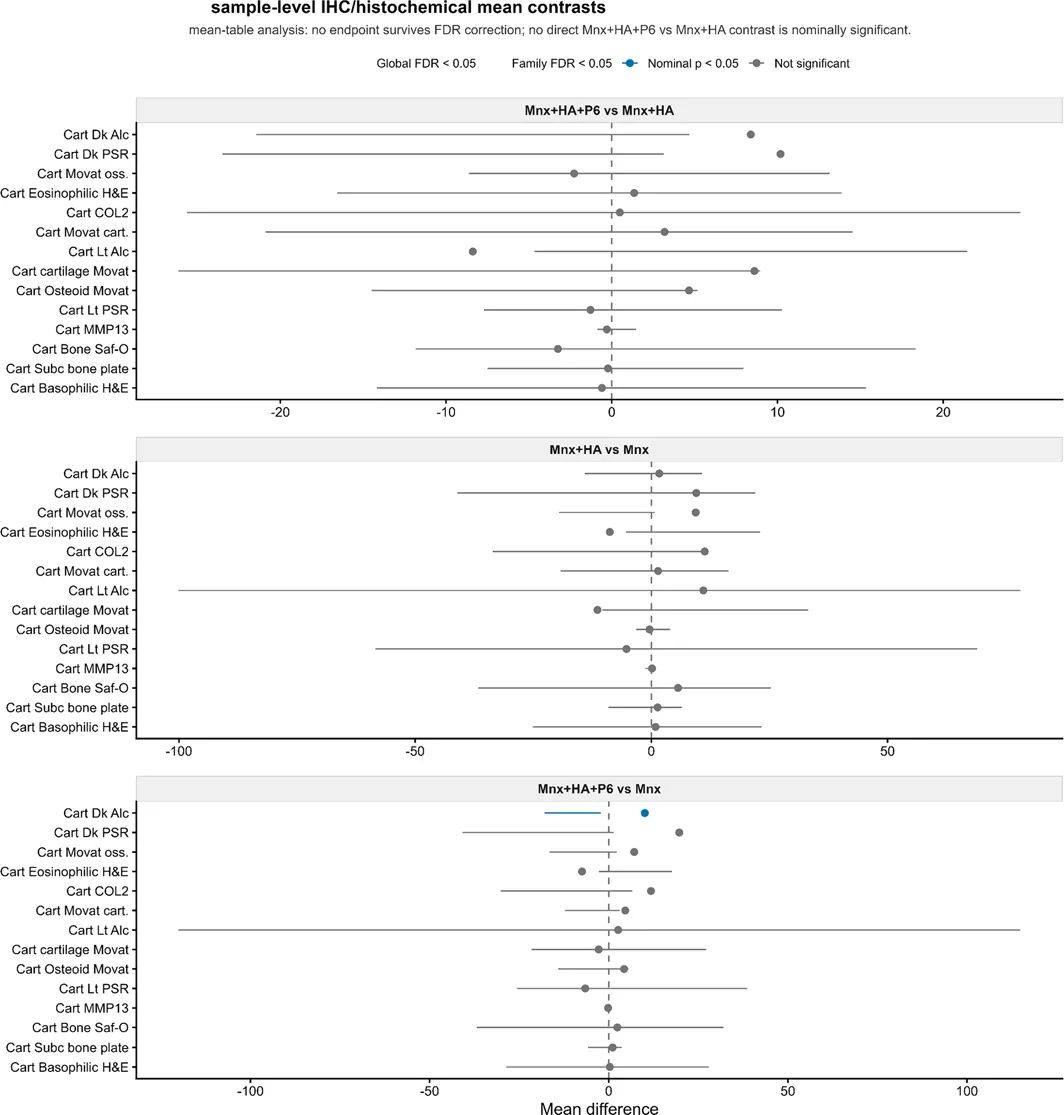
Sample‐level IHC/histochemical mean contrasts. Forest plot of sample‐level IHC/histochemical mean contrasts for selected cartilage‐region endpoints. Points indicate estimated mean differences, and horizontal bars indicate 95% confidence intervals. The dashed vertical line denotes no estimated group difference. Facets show the primary biological contrast (Mnx + HA + P6 vs. Mnx + HA) and injury‐reference contrasts (Mnx + HA vs. Mnx and Mnx + HA + P6 vs. Mnx). No endpoint survived false‐discovery‐rate correction in this sample‐level mean analysis, and no direct Mnx + HA + P6 versus Mnx + HA contrast reached nominal significance. The only nominal signal shown in this summary occurred outside the primary contrast and is therefore interpreted as exploratory. Endpoint labels are abbreviated for readability: Cart, cartilage region; Dk, dark color‐fraction component; Lt, light color‐fraction component; PSR, Picrosirius Red; Alc, Alcian Blue; Saf‐O, Safranin O; COL2, type II collagen; Subc bone plate, subchondral bone plate. This analysis is reported separately from the broader animal/knee‐level IHC/histomorphometry mixed‐model screen.

IHC and histochemical outcomes were analyzed as exploratory tissue‐pattern readouts (Figure 8; Supporting Information S1: Tables S5–S8). The updated sample‐level IHC/histochemical mean results did not show robust group‐level separation. No IHC/histochemical mean endpoint survived FDR correction, and no direct Mnx + HA + P6 versus Mnx + HA contrast reached nominal significance in this analysis. The smallest direct primary‐contrast signal occurred for cartilage Dark Picrosirius Red, but it remained non‐significant and was not FDR‐supported.

### 3.6 Corrected Animal/Knee‐Level Contrast Analysis

Corrected animal/knee‐level analyses for the prespecified primary and key secondary endpoints are summarized in Figures 7 and 8, Table 2, and Supporting Information S1: Tables S4, S11 and S12. The primary biological contrast was Mnx + HA + P6 versus Mnx + HA, while treatment‐versus‐injury comparisons (Mnx + HA vs. Mnx and Mnx + HA + P6 vs. Mnx) and comparisons with Intact were used for contextual interpretation.

For the primary structural endpoint, percent trabecular bone volume/BV/TV, the direct Mnx + HA + P6 versus Mnx + HA contrast was small and imprecise (+1.52% points; 95% CI −2.22 to 5.26; *p* = 0.427; family *q* = 0.762). This does not support a robust added P6 effect beyond the HA carrier. In contrast, treatment‐versus‐injury comparisons showed directionally favorable trabecular patterns. Mnx + HA + P6 was nominally higher than Mnx for BV/TV (+4.75% points; 95% CI 0.13 to 9.37; *p* = 0.044), although this did not survive family‐level or global FDR correction. Mnx + HA also showed a positive, but non‐significant, BV/TV estimate versus Mnx (+3.23% points; 95% CI −1.39 to 7.85; *p* = 0.170).

Other trabecular microCT endpoints supported the same cautious interpretation. Several contrasts showed structural differences, particularly for trabecular separation and trabecular number, but the direction and multiplicity context did not establish consistent peptide‐specific superiority. For trabecular separation, Mnx + HA showed a lower value than Mnx, consistent with a favorable treatment‐versus‐injury pattern, whereas the direct Mnx + HA + P6 versus Mnx + HA contrast did not support an added peptide benefit. Because lower trabecular separation is generally interpreted as favorable in this context, statistically significant contrasts for this endpoint were interpreted together with their biological direction and not only by *p* value. Cortical/subchondral and other microCT endpoints were treated as exploratory structural readouts and did not provide consistent corrected evidence that Mnx + HA + P6 outperformed Mnx + HA.

Histological summary endpoints in Figure 3 supported the same cautious interpretation: selected treatment‐versus‐Mnx tissue patterns were observed, but the direct Mnx + HA + P6 versus Mnx + HA histological contrasts did not establish additive peptide benefit.

Overall, Figure 3 shows that the strongest evidence lies in treatment‐associated structural and subchondral patterns relative to Mnx, whereas the direct Mnx + HA + P6 versus Mnx + HA contrast remains weak across the primary and key secondary endpoints. The corrected analysis therefore supports HA‐associated trabecular microCT architecture after meniscectomy, with selected supportive histological patterns, but does not demonstrate consistent peptide‐specific superiority.

## 4 Discussion

The present findings are best read as a controlled large‐animal biomaterials study and not as a definitive OA efficacy trial. HA‐based intra‐articular treatment was associated with a more favorable trabecular microarchitectural pattern after surgically induced meniscal injury‐associated OA‐like change. Because the study used a single terminal 12‐week endpoint and did not include longitudinal imaging, the data cannot determine whether this pattern reflects prevention of injury‐associated deterioration, partial structural preservation, altered remodeling, or recovery after injury. The incremental value of P6 over HA was less consistent. That distinction is important because treatment‐versus‐injury patterns cannot be used as evidence of peptide‐specific superiority unless the direct Mnx + HA + P6 versus Mnx + HA contrast supports that interpretation.

The ovine stifle remains a useful model for translational intra‐articular biomaterials. It permits clinically realistic surgical handling, arthrocentesis, microCT, compartment‐specific histology, and combined assessment of cartilage, meniscus, synovium, and subchondral bone within a mechanically loaded joint [44, 51, 52]. The model also has limits. Spontaneous OA is not a reliable disease model in sheep, and surgically induced degeneration should be described as post‐traumatic OA‐like change rather than naturally occurring human OA [44]. Meniscal injury or meniscectomy provides a more consistent post‐traumatic phenotype than ligament transection alone in sheep and goats [14, 15, 44]. The present model, therefore, maps most closely to early meniscal injury–associated joint degeneration.

This model is clinically relevant because PTOA often affects younger, physically active individuals, including athletes, military personnel, and those in physically demanding work [10, 53, 54]. Joint‐preserving treatments have particular value in this setting, since early arthroplasty carries a higher lifetime revision burden than arthroplasty performed later in life [55, 56]. Therefore, a material that could attenuate early tissue‐level changes after meniscal injury would address a meaningful clinical problem. The present data do not prove such disease modification, but they support continued evaluation of cross‐linked HA‐based intra‐articular hydrogels in this context.

The synovial component of the model deserves attention even though the synovium was not directly evaluated here. Ovine meniscectomy produces chronic synovial pathology characterized predominantly by subintimal fibrosis and hypervascularity, with less prominent inflammatory‐cell infiltration [18, 19]. Previous ovine work has shown that intra‐articular hyaluronan can reduce synovial vascularity and fibrosis‐related pathology [18, 19]. This is relevant because HA‐based materials are not simply mechanical lubricants. Their performance may depend on their interactions with the synovium, inflammatory mediators, and the osteochondral unit.

Therefore, the HA component should be discussed as a biologically active material. Mechanistic studies of intra‐articular HA support actions involving CD44‐mediated signaling, suppression of IL‐1β‐driven catabolic pathways, reduced MMP and ADAMTS expression, modulation of iNOS and PGE2, stimulation of proteoglycan and glycosaminoglycan synthesis, improved lubrication, and nociceptive signaling [20, 22]. Crosslinking and molecular weight further influence residence time, viscoelastic behavior, degradation profile, and biological activity [21, 23]. In a rat PTOA model, cross‐linked high‐molecular‐weight HA produced better functional and structural outcomes than linear HA [26]. This supports the interpretation of the present material as an engineered HA hydrogel rather than a generic viscosupplement.

The peptide component needs more restrained interpretation. P2 and P6 are proline‐rich, intrinsically disordered sequences inspired by amelogenin and related mineralized‐tissue proteins [33, 34, 35]. The peptide family has shown bioactivity in wound healing, collagen stimulation, mineral nucleation, osteogenic differentiation, and bone regeneration [33, 34, 35]. Amelogenin itself has recently been linked to cartilage and osteochondral repair biology [36, 37]. These observations justify evaluating P6 in the joint, but they do not justify a direct cartilage‐regeneration claim.

This caution is essential because bone regeneration and articular cartilage preservation are not the same biological problem. Mineral nucleation and osteoinduction may be beneficial in bone defects, but uncontrolled mineralization within articular cartilage could promote hypertrophy, calcification, or osteophyte‐like pathology. Because HA + P6 was delivered intra‐articularly as a cross‐linked HA formulation, the present study does not establish diffusion of P6 to mineralized subchondral bone or direct peptide exposure in that compartment. A more conservative interpretation is that any P6‐related signal, if present, would most plausibly arise through indirect modulation of the osteochondral joint environment, including matrix turnover, inflammatory regulation, synovial‐ or cartilage‐mediated signaling, and subsequent subchondral remodeling. Prior HA + P6 soft‐tissue data showing reduced TNF‐α and CD80‐associated inflammatory macrophage signaling provide a plausible basis for this hypothesis [38]. The current sheep data, however, does not directly prove this mechanism.

The findings also fit within a broader movement toward injectable, bioactive, mechanically compatible intra‐articular biomaterials. Recent large‐animal work with injectable supramolecular‐covalent scaffolds has shown that cartilage repair in mechanically active joints requires more than biological potency alone: the material must remain localized, tolerate load, integrate with tissue, and support hyaline‐like matrix formation [32]. Compared with morphogen‐delivery strategies, HA + P6 is a more restrained concept, but the same translational principles apply. Future studies should therefore measure not only tissue endpoints but also material persistence, pain registration, synovial response, and mechanical function.

The limitations of this study are substantial and should remain visible. This was a controlled pilot study in large animals and not a confirmatory efficacy study. The design was not powered to establish the superiority of Mnx + HA + P6 over Mnx + HA, and the corrected animal‐ and knee‐level analyses appropriately reduced the strength of the claims. Although Intact no‐surgery/no‐injection knees were included as baseline reference tissue, they were not part of a randomized treatment arm, and endpoint coverage differed across microCT, histology, and IHC/histochemical analyses. The Mnx group did not receive saline or vehicle injections; therefore, injection‐related effects cannot be separated from material‐related effects. The study also included only one 12‐week endpoint, no synovial histology, no tibial plateau microCT outcome, and no pain, gait, or other functional readout. The injection schedule was not independently optimized for intra‐articular residence time, peptide release, or the timing of biological response. These limitations also define how the findings should be interpreted. The present dataset does not provide comprehensive evidence for clinical translation, does not establish disease modification, and should not be used to suggest a change in practice. The strongest evidence comes from trabecular microCT patterns associated with HA‐based treatment following meniscectomy. The contribution of P6 should therefore be interpreted cautiously. Mnx + HA + P6 showed selected exploratory matrix‐associated signals, including the Dark Picrosirius Red finding, but the direct Mnx+ HA + P6 versus Mnx + HA comparison did not show a consistent peptide‐specific advantage across the primary structural, histological, and IHC/histochemical endpoints. Accordingly, the exploratory Dark Picrosirius Red signal is best interpreted as a collagen‐associated matrix‐pattern observation rather than direct evidence of collagen maturation, cartilage repair, or peptide‐specific disease modification. The data support further investigation of the cross‐linked HA platform and indicate that the present P6‐supplemented formulation has not yet been validated as an improvement over Mnx + HA.

A useful outcome of this pilot is a framework for determining whether HA + P6 should advance as an intra‐articular biomaterial candidate. A confirmatory study should prospectively define a small number of animal/knee‐level primary endpoints, include both no‐surgery reference tissue and vehicle‐injected meniscectomy controls, extend follow‐up beyond 12 weeks, include synovial and tibial‐plateau outcomes, add gait or pain‐related measures where feasible, and directly measure material persistence, peptide retention/release, and local tissue exposure in the joint environment. It should also be powered around the direct Mnx + HA + P6 versus Mnx + HA comparison, since this is the only contrast that can determine whether P6 adds value beyond the HA carrier. Until such data are available, the appropriate conclusion is that cross‐linked HA‐based intra‐articular treatment produced favorable structural patterns that merit follow‐up, whereas peptide‐specific benefit remains exploratory.

## 5 Conclusions

In this sheep model of surgically induced partial lateral meniscectomy‐associated early OA‐like change, cross‐linked HA‐based intra‐articular treatment was associated with favorable trabecular microarchitectural changes compared with the Mnx meniscectomy‐only injury reference group. Histological scoring and most image‐based histochemical and immunohistochemical endpoints showed limited treatment separation, indicating that the structural microCT findings were not broadly confirmed by cartilage or molecular tissue readouts. HA + P6 showed selected matrix‐associated signals, but the current dataset does not demonstrate consistent superiority over HA alone. These findings support further investigation of cross‐linked HA‐based intra‐articular hydrogels in early post‐traumatic joint degeneration, while the additive value of P6 should be tested in a focused confirmatory study with prespecified animal‐ and knee‐level endpoints, injection controls, longitudinal follow‐up, and appropriate multiplicity control.

## Author Contributions

Concept and design: H.J.H., S.P.L., Ø.Ø., A.D.L., T.E.K., F.M., and A.G.C. Animal procedures and study logistics: M.P., F.M., A.G.C., A.M.S., and H.J.H. MicroCT, histology, immunohistochemistry and image analysis: A.M.S., R.J., S.S., M.R., L.P.N., T.E.K., and H.J.H. Data interpretation: A.M.S., R.J., S.S., M.R., L.P.N., T.E.K., Ø.Ø., A.D.L., S.P.L., and H.J.H. Drafting: A.M.S. and H.J.H. Critical revision and final approval: all authors. H.J.H. takes responsibility for the integrity of the work as a whole.

## Ethics Statement

All animal procedures were approved by the Ethical Committee of the Rof Codina Foundation and the regional government of Xunta de Galicia (approval number 03/18/LU‐001) and complied with applicable animal‐welfare regulations.

## Consent

The authors have nothing to report.

## Conflicts of Interest

S.P.L., H.J.H., Ø.Ø., and A.D.L. are shareholders of Regenarti AS, which owns intellectual property related to the HA + P6 formulation. The remaining authors declare no competing interests related to this work.

## Supporting information


Supporting Information S1

## Data Availability

The data that support the findings of this study are available on request from the corresponding author. The data are not publicly available due to privacy or ethical restrictions.
