# Supplementary material for: Limited Added Effect of Proline‐Rich P6 Peptide to Cross‐Linked Hyaluronic Acid After Partial Lateral Meniscectomy in Sheep
Source: Smart Med. 2026 Aug 24;5(4):e70048. doi: 10.1002/smmd.70048 (PMC13517024; doi:10.1002/smmd.70048)
Supplement: Supplementary file 1 [file SMMD-5-e70048-s001.docx]

**Supplementary Material**

**Limited Added Effect of Proline-Rich P6 Peptide to Crosslinked Hyaluronic Acid after Partial Lateral Meniscectomy in Sheep**

This Supporting Information file contains representative histology, histochemical and immunohistochemical panels (Supplementary Figures S1–S6), supplementary statistical-methods detail, endpoint-specific modelability and sensitivity analyses (Supplementary Figure S7; Supplementary Tables S2, S3 and S8), exploratory integrated and mixed-model summaries (Supplementary Figures S8 and S9; Supplementary Tables S6, S7 and S9), the future-study power/precision simulation (Supplementary Figure S10; Supplementary Table S10), and the complete newly generated tables used to update the main manuscript (Supplementary Tables S1–S14). All analyses use the corrected four-group nomenclature: Intact, Mnx, Mnx+HA, and Mnx+HA+P6. In this nomenclature, Intact refers to no-surgery/no-injection knees, Mnx refers to meniscectomy-only injury knees, Mnx+HA refers to meniscectomy followed by crosslinked hyaluronic acid treatment, and Mnx+HA+P6 refers to meniscectomy followed by crosslinked hyaluronic acid containing the proline-rich P6 peptide.

The corrected generated dataset comprises 7 Intact no-surgery/no-injection knees, 4 Mnx knees, 6 Mnx+HA knees, and 6 Mnx+HA+P6 knees. Source-data provenance and endpoint-family allocation are documented in Supplementary Tables S1 and S2. Because endpoint availability differed across histology, immunohistochemistry/histochemistry and microCT datasets, all analyses were preceded by endpoint-specific coverage checks, summarized in Supplementary Figure S7 and Supplementary Table S3. The Prism-updated immunohistochemical and histochemical analyses used knee/sample-level mean endpoints for the principal supplementary comparisons (Supplementary Table S5). A broader mixed-model immunohistochemistry/histomorphometry screen was retained separately as an exploratory context (Supplementary Figure S9; Supplementary Tables S6 and S7) and was not treated as confirmatory evidence of peptide-specific efficacy.

**Supplementary Methods**

**Supplementary statistical analyses**

**Data correction, group nomenclature and experimental unit**

All supplementary statistical analyses were repeated after correction of the group labels and source-data structure. The corrected four-group nomenclature was Intact, Mnx, Mnx+HA, and Mnx+HA+P6. The refreshed source-data provenance and endpoint-family allocation are documented in Supplementary Tables S1 and S2. The main biological contrast of interest was Mnx+HA+P6 versus Mnx+HA, testing whether addition of the proline-rich P6 peptide conferred benefit beyond crosslinked hyaluronic acid alone; the corresponding primary and key secondary contrasts are summarized in Supplementary Table S4.

The animal was treated as the primary experimental clustering unit. Lower-level observations, including sections, microscopic fields, regions of interest, image-analysis exports and repeated technical rows, were not treated as independent biological replicates. For the primary supplementary analyses, these lower-level observations were aggregated to knee/sample-level endpoint values before inferential testing. This approach was used to reduce pseudoreplication and to ensure that the reported sample size reflected biological rather than technical replication. Endpoint-family-specific animal/knee counts are reported in Supplementary Table S2, with modelability summarized in Supplementary Table S3.

**Endpoint coverage and modelability audit**

Endpoint-specific coverage was audited before modeling. Endpoints were considered modelable only when the available observations allowed estimation of the relevant group effects and, where applicable, the animal-level random intercept. The endpoint-family modelability gate is visualized in Supplementary Figure S7 and tabulated in Supplementary Table S3.

The H&E histology dataset contained 12 modelable endpoints, with coverage across 9 animals and 17 knees. The immunohistochemical and histochemical sample-level mean dataset contained 28 modelable endpoints, with coverage across 10 animals and 18 samples/knees. The microCT cortical and surface dataset contained modelable endpoints across 12 animals and 23 knees/samples. MicroCT trabecular endpoints had more limited and variable coverage; Percent Trabecular Bone Volume was modelable with 10 animals and 17 knees/samples. These endpoint-specific counts and their implications are reported in Supplementary Table S3 and illustrated in Supplementary Figure S7. Endpoints with inadequate coverage, non-estimable group effects or unstable model fits were flagged and were not used for confirmatory interpretation.

**Primary and secondary contrasts**

The prespecified primary contrast was:

**Mnx+HA+P6 versus Mnx+HA**

This contrast directly tested whether the P6 peptide provided measurable benefit beyond that of crosslinked hyaluronic acid alone. The primary structural contrast and selected secondary contrasts used for manuscript interpretation are reported in Supplementary Table S4.

Additional contrasts were estimated to contextualize the injury and treatment response, including comparisons of Mnx with Intact and treatment groups with Mnx, where model coverage permitted. These additional contrasts were interpreted as secondary or contextual and were not used to claim peptide-specific efficacy unless they supported the primary Mnx+HA+P6-versus-Mnx+HA comparison. Key contextual contrasts are summarized in Supplementary Table S4, while the exploratory IHC/histomorphometry contrasts are reported separately in Supplementary Table S7 and Supplementary Figure S9.

**Primary modeling framework**

For each endpoint, a linear mixed model with an animal-level random intercept was fitted when the data structure made the random effect identifiable. The fixed effect was the treatment group. When the animal-level random intercept could not be reliably estimated, for example because only one knee/sample per animal was available for a given endpoint or because of sparse group coverage, an animal/knee-level linear model was used instead, and the endpoint was flagged accordingly. The endpoint-family modelability summary is provided in Supplementary Table S3.

Estimated marginal means and prespecified pairwise contrasts were obtained for all modelable endpoints. Effect estimates are reported with 95% confidence intervals, nominal p values and multiplicity-adjusted q values. The primary/key secondary results are summarized in Supplementary Table S4, the strict IHC/histochemical mean analysis in Supplementary Table S5, the exploratory IHC/histomorphometry screen in Supplementary Tables S6 and S7, and the sensitivity-analysis overview in Supplementary Table S8. The direction and magnitude of the effect estimate were considered alongside the adjusted statistical evidence, rather than relying on nominal p values alone.

**Multiplicity control**

Multiplicity was controlled using the Benjamini–Hochberg false-discovery-rate procedure. Adjustment was performed within endpoint families and also globally across the broader set of modeled endpoints. Endpoint families included histological scores, immunohistochemical/histochemical mean endpoints, exploratory immunohistochemistry/histomorphometry endpoints, cortical and surface microCT endpoints, and trabecular microCT endpoints, where applicable. Family and global q values are reported in the endpoint result tables where relevant (Supplementary Tables S4, S6 and S7).

Results were interpreted hierarchically. Endpoints surviving global FDR correction were considered the strongest statistical signals. Endpoints surviving only family-level correction were considered supportive within that endpoint family. Nominally significant endpoints that did not survive FDR correction were interpreted as exploratory and hypothesis-generating only. This evidence hierarchy is particularly relevant for reconciling the strict IHC sample-level analysis with the broader exploratory IHC/histomorphometry screen (Supplementary Tables S5–S7; Supplementary Figure S9).

**H&E histology sensitivity analyses**

For H&E histology, the primary analysis used aggregated knee-level endpoint values. Representative histological panels are shown in Supplementary Figure S1, with articular cartilage close-up histology in Supplementary Figure S5. Because several H&E outcomes were ordinal scores, cumulative-link ordinal models were also fitted as sensitivity analyses where model coverage permitted (Supplementary Table S8). These ordinal models were used to examine whether the conclusions depended on treating the scores as approximately continuous. The ordinal sensitivity results did not provide consistent evidence that Mnx+HA+P6 improved H&E-derived joint pathology relative to Mnx+HA.

Leave-one-animal-out influence checks were also performed for H&E endpoints to determine whether apparent effects were driven by single animals. Endpoints showing unstable estimates or direction changes after removal of individual animals were interpreted cautiously. These robustness checks are summarized together with the other sensitivity analyses in Supplementary Table S8.

**Immunohistochemical and histochemical sample-level mean analyses**

The updated Prism/R immunohistochemical and histochemical analyses were based on sample-level mean endpoints. Representative IHC/histochemical panels are shown in Supplementary Figures S2–S4, with articular cartilage collagen-associated close-up panels in Supplementary Figure S6. This sample-level mean analysis was used as the principal supplementary analysis for the revised immunohistochemical and histochemical results because it aligned the unit of analysis with the biological sample/knee and avoided treating multiple image fields or export rows as independent replicates. The strict sample-level analysis summary is provided in Supplementary Table S5.

For bounded proportional or percentage-type endpoints, fractional or beta-model sensitivity analyses were performed where technically appropriate (Supplementary Table S8). These analyses tested whether conclusions were sensitive to the distributional assumptions of the linear model. The sample-level mean analysis remained largely negative for correction. No omnibus immunohistochemical or histochemical endpoint survived FDR correction, and no Mnx+HA+P6-versus-Mnx+HA sample-level mean endpoint reached nominal significance (Supplementary Table S5).

**Exploratory immunohistochemistry/histomorphometry mixed-model screen**

In addition to the sample-level mean analysis, a broader immunohistochemistry/histomorphometry mixed-model screen was performed as an exploratory context. This screen made fuller use of the available image-analysis structure, while, where possible, accounting for animal-level clustering. Because the screen involved a larger set of endpoints and a more complex lower-level data structure, it was not treated as the primary basis for claims of treatment efficacy. The relationship between the strict sample-level mean analysis and the broader mixed-model screen is summarized in Supplementary Table S6.

In this exploratory screen, cartilage Dark Picrosirius Red showed a positive Mnx+HA+P6-versus-Mnx+HA signal, with an estimated difference of +11.69 percentage points, 95% CI 5.32 to 18.06, p = 0.00032, global q = 0.0106 and family q = 0.0537 (Supplementary Table S7; Supplementary Figure S9). This result was globally FDR-supported but narrowly non-significant after correction for the immunohistochemistry family FDR. It was therefore retained as an exploratory collagen-associated matrix signal only. It was not interpreted as confirmatory evidence that P6 confers a consistent added therapeutic benefit over crosslinked hyaluronic acid alone.

**MicroCT analyses**

MicroCT endpoints were analyzed separately for the cortical/surface and trabecular compartments because endpoint coverage and biological interpretation differed between them. Cortical and surface endpoints were modelable across 12 animals and 23 knees/samples. Trabecular endpoints had more variable coverage, and only endpoints with sufficient data structure were modeled. Percent Trabecular Bone Volume was modelable with 10 animals and 17 knees/samples. MicroCT modelability is summarized in Supplementary Table S3 and Supplementary Figure S7; the primary structural contrast is reported in Supplementary Table S4, and the canonical key microCT structural endpoint results are summarized in Supplementary Table S11.

Linear mixed models with animal-level random intercepts were fitted where identifiable; otherwise, animal/knee-level linear models were used and flagged. Heteroscedastic generalized least-squares sensitivity analyses were performed on selected microCT endpoints to assess whether unequal variance across groups materially affected the conclusions. Normal-approximation Bayesian sensitivity analyses were also performed for microCT endpoints to assess uncertainty in estimated group differences under small-sample conditions. These analyses are summarized in Supplementary Table S8, with compact primary-contrast robustness outputs in Supplementary Table S12, and did not provide robust evidence of a consistent Mnx+HA+P6 benefit over Mnx+HA. The implications for future confirmatory study design are shown in Supplementary Figure S10 and summarized in Supplementary Table S10.

**Robustness and influence analysis**

Several additional sensitivity analyses were performed across endpoint families to assess robustness under small-sample and unbalanced-data conditions. Animal-cluster bootstrap analyses were used to estimate the stability of treatment effects while preserving the animal as the resampling unit. Animal-level permutation analyses were used as distribution-light checks of the treatment contrasts where endpoint coverage permitted. Leave-one-animal-out analyses were used to identify contrasts that were disproportionately influenced by individual animals. The full sensitivity analysis overview is provided in Supplementary Table S8, and the compact key-endpoint bootstrap/permutation robustness summary is provided in Supplementary Table S12.

The sensitivity analyses were interpreted as robustness checks rather than independent confirmatory tests. Signals that were not stable across sensitivity analyses, or that depended on a single animal or narrow modeling assumption, were treated as exploratory. This interpretation is reflected in the sensitivity analysis summary in Supplementary Table S8.

**Direction-aligned exploratory repair score**

An exploratory direction-aligned repair score was generated to summarize whether multiple endpoints changed in a direction consistent with tissue preservation or repair. The score was intended as a descriptive integration of endpoint directionality and was not used as a validated composite outcome. Because the score combines heterogeneous endpoints with different biological meanings, it was interpreted only as exploratory support for pattern recognition and not as evidence of treatment efficacy. The score distribution is shown in Supplementary Figure S8, and the integrated repair-score contrasts are reported in Supplementary Table S9.

**Interpretation of the revised statistical evidence**

The revised analysis consistently indicates that the dataset does not provide robust evidence that adding P6 confers a consistent therapeutic advantage over crosslinked hyaluronic acid alone after partial lateral meniscectomy in sheep. The primary and key secondary endpoint results are summarized in Supplementary Table S4. The strongest exploratory signal was observed for cartilage Dark Picrosirius Red in the broader immunohistochemistry/histomorphometry mixed-model screen (Supplementary Table S7; Supplementary Figure S9). However, this finding was not supported by nominal significance in the updated sample-level mean Mnx+HA+P6-versus-Mnx+HA analysis (Supplementary Table S5) and did not survive immunohistochemistry-family FDR correction (Supplementary Table S7). It is therefore best interpreted as a hypothesis-generating signal from a collagen-associated matrix.

Overall, the supplementary statistical analyses support a conservative interpretation: crosslinked hyaluronic acid with P6 did not show a consistent added benefit over crosslinked hyaluronic acid alone across histology, immunohistochemistry/histochemistry and microCT endpoints (Supplementary Tables S4–S8). Representative tissue-level context is shown in Supplementary Figures S1–S6, endpoint coverage and modelability in Supplementary Figure S7 and Supplementary Tables S2 and S3, the exploratory integrated repair score in Supplementary Figure S8 and Supplementary Table S9, and future power/precision implications in Supplementary Figure S10 and Supplementary Table S10. Given the small and unbalanced group sizes, particularly the Mnx group, the absence of FDR-supported differences should not be interpreted as proof of equivalence. The revised statistical evidence instead indicates that any peptide-specific effect, if present, was not sufficiently consistent or robust to support a strong claim of efficacy in this dataset.

# Supplementary Figures

Supplementary Figure S1. Representative histological staining in the A1 anteroposterior view. Panels show matched sections from Intact, Mnx, Mnx+HA, and Mnx+HA+P6 groups stained with H&E, Movat pentachrome, and Picrosirius Red. These panels illustrate orientation-specific tissue context and are not independent biological replicates.

Supplementary Figure S2. Representative histochemical and immunohistochemical staining in the A1 anteroposterior view. Panels show Intact, Mnx, Mnx+HA, and Mnx+HA+P6 sections stained for Alcian Blue, type II collagen (COL2), MMP13, and Safranin O.

Supplementary Figure S3. Representative histochemical and immunohistochemical staining in the A2 lateral view. Panels show Intact, Mnx, Mnx+HA, and Mnx+HA+P6 sections stained for Alcian Blue, COL2, MMP13, and Safranin O.

Supplementary Figure S4. Representative histochemical and immunohistochemical staining in the B1 axial view. Panels show Intact, Mnx, Mnx+HA, and Mnx+HA+P6 sections stained for Alcian Blue, COL2, MMP13, and Safranin O. The axial view is included as anatomical context, not as an independent replicate.

**Supplementary Figure S5.** Articular cartilage close-up histology. Representative high-magnification crops of the articular cartilage surface from the B1 orientation, shown across Intact, Mnx, Mnx+HA, and Mnx+HA+P6 groups. H&E staining highlights overall cartilage morphology, surface integrity, cellular organization, and subchondral tissue architecture. Movat pentachrome staining illustrates cartilage matrix architecture, proteoglycan-rich regions, collagenous tissue, and the morphology of the osteochondral interface.

**Supplementary Figure S6.** Articular cartilage collagen-associated close-up panel. Representative high-magnification crops of the articular cartilage surface from the B1 orientation, shown across Intact, Mnx, Mnx+HA, and Mnx+HA+P6 groups. Type II collagen immunostaining (COL2) is shown to illustrate cartilage-associated collagen matrix localization, while Picrosirius Red staining provides complementary visualization of collagen-rich matrix architecture in the articular cartilage and osteochondral region.

**Supplementary Figure S7.** Endpoint-specific modelability gate. The bar chart summarizes the number of modelable endpoint definitions by endpoint family after source-data harmonization and endpoint-specific coverage auditing. The figure illustrates why the manuscript does not use a single global group size across microCT, H&E, and IHC/histochemical outcomes; the inferential tables report endpoint-specific counts of animals/knees.

**Supplementary Figure S8.** Exploratory direction-aligned repair score. Endpoints with defensible favorable directions were standardized so that higher z-scores indicate a more favorable repair-associated pattern. The score is exploratory and does not replace endpoint-level inference; the Mnx+HA+P6-versus-Mnx+HA contrast did not provide evidence of added peptide benefit.

**Supplementary Figure S9.** Exploratory mixed-model signals from immunohistochemical and histochemical endpoints**.** Forest plot showing nominally significant (p < 0.05) group contrasts identified in the exploratory animal/knee-level linear mixed-model analysis of IHC/histochemical endpoints. Points indicate estimated group differences, and horizontal lines indicate 95% confidence intervals. The vertical dashed line marks no difference (0). Blue points denote contrasts with nominal p < 0.05 only, whereas red points denote contrasts that also remained significant after global false-discovery-rate (FDR) correction. Among the displayed signals, cartilage Dark Picrosirius Red (Cart Dk PSR), Mnx+HA+P6 versus Mnx+HA, was the only contrast supported after global FDR correction. All other contrasts displayed were nominal only and should be interpreted as exploratory. Abbreviations: Intact, no-surgery/no-injection control; Mnx, meniscectomy only; Mnx+HA, meniscectomy + crosslinked hyaluronic acid; Mnx+HA+P6, meniscectomy + crosslinked hyaluronic acid with P6 peptide; Saf-O, Safranin O; PSR, Picrosirius Red.

**Supplementary Figure S10.** Monte Carlo power/precision simulation for the future Mnx+HA+P6-versus-Mnx+HA contrast. The simulation illustrates the sample-size implications of detecting plausible effects in key trabecular microCT endpoints. The current pilot should therefore be interpreted as hypothesis-generating and as guidance for the design of a confirmatory study, not as a powered efficacy study.

# Supplementary Tables

The tables below present the detailed statistical audit and analysis outputs that support the revised manuscript. All tables use the final labels Intact, Mnx, Mnx+HA, and Mnx+HA+P6. Endpoint-specific animal/knee counts are reported because microCT, histology, and IHC/histochemical endpoint families have different coverage. Source provenance is reported in Supplementary Table S1, allocation and modelability in Supplementary Tables S2 and S3, primary/key results in Supplementary Table S4, IHC/histochemical analyses in Supplementary Tables S5–S7, sensitivity analyses in Supplementary Table S8, exploratory repair-score contrasts in Supplementary Table S9, and power/precision simulations in Supplementary Table S10. The added compact audit tables report canonical key microCT endpoints, primary-contrast robustness, source/unit harmonization, and the machine-readable output index in Supplementary Tables S11-S14.

**Supplementary Table S1.** Refreshed source-data provenance.

| **Source file** | **Exists** | **Size** | **Modified time** |
| --- | --- | --- | --- |
| C:/R/Knee/Sheep_Knee_All_Data_R_Friendly.xlsx | Yes | 506,407 bytes | 2026-06-15 20:26:50 |
| C:/R/Knee/Sheep_Knee_All_Data_R_Friendly.csv | Yes | 1,282,265 bytes | 2026-06-15 20:26:48 |
| C:/R/Knee/data_processed/sheep_knee_all_data_histo_ihc_microCT_R_friendly_latest.xlsx | Yes | 506,407 bytes | 2026-06-15 20:26:50 |
| C:/R/Knee/data_processed/endpoint_group_coverage_vs_trabecular_latest.xlsx | Yes | 22,646 bytes | 2026-06-15 20:29:39 |
| C:/R/Knee/data_processed/endpoint_group_coverage_vs_trabecular_latest.csv | Yes | 61,171 bytes | 2026-06-15 20:29:39 |

*Source table: refreshed_data_provenance_audit.csv. These R-friendly files were treated as the authoritative data sources for the final refreshed analysis.*

**Supplementary Table S2.** Endpoint-family-specific allocation audit.

| **Endpoint family** | **Group** | **N animals** | **N knees/samples** | **Sample IDs** |
| --- | --- | --- | --- | --- |
| HE_histology_score | Intact | 1 | 1 | 162_24 |
| HE_histology_score | Mnx | 4 | 4 | 140_24, 141_24, 144_24, 155_24 |
| HE_histology_score | Mnx+HA | 6 | 6 | 139_24, 142_24, 152_24, 153_24, 166_24, 167_24 |
| HE_histology_score | Mnx+HA+P6 | 6 | 6 | 143_24, 151_24, 154_24, 156_24, 165_24, 168_24 |
| IHC_histomorphometry | Intact | 2 | 2 | 148_24, 162_24 |
| IHC_histomorphometry | Mnx | 4 | 4 | 140_24, 141_24, 144_24, 155_24 |
| IHC_histomorphometry | Mnx+HA | 6 | 6 | 139_24, 142_24, 152_24, 153_24, 166_24, 167_24 |
| IHC_histomorphometry | Mnx+HA+P6 | 6 | 6 | 143_24, 151_24, 154_24, 156_24, 165_24, 168_24 |
| MicroCT_cortical | Intact | 5 | 9 | 145_24, 146_24, 147_24, 148_24, 149_24, 150_24, 162_24, 163_24, 164_24 |
| MicroCT_cortical | Mnx | 3 | 3 | 141_24, 144_24, 155_24 |
| MicroCT_cortical | Mnx+HA | 5 | 5 | 142_24, 152_24, 153_24, 166_24, 167_24 |
| MicroCT_cortical | Mnx+HA+P6 | 6 | 6 | 143_24, 151_24, 154_24, 156_24, 165_24, 168_24 |
| MicroCT_surface | Intact | 5 | 9 | 145_24, 146_24, 147_24, 148_24, 149_24, 150_24, 162_24, 163_24, 164_24 |
| MicroCT_surface | Mnx | 3 | 3 | 141_24, 144_24, 155_24 |
| MicroCT_surface | Mnx+HA | 5 | 5 | 142_24, 152_24, 153_24, 166_24, 167_24 |
| MicroCT_surface | Mnx+HA+P6 | 6 | 6 | 143_24, 151_24, 154_24, 156_24, 165_24, 168_24 |
| MicroCT_trabecular | Intact | 4 | 7 | 145_24, 146_24, 147_24, 148_24, 149_24, 150_24, 162_24 |
| MicroCT_trabecular | Mnx | 2 | 2 | 141_24, 144_24 |
| MicroCT_trabecular | Mnx+HA | 4 | 4 | 142_24, 153_24, 166_24, 167_24 |
| MicroCT_trabecular | Mnx+HA+P6 | 4 | 4 | 143_24, 154_24, 156_24, 168_24 |

*Source table: allocation_audit.csv. Counts are endpoint-family specific and should not be replaced by a single global n.*

**Supplementary Table S3.** Endpoint-specific modelability summary by endpoint family.

| **Endpoint family** | **Endpoints audited** | **Modelable endpoints** | **Complete four-group endpoints** | **Typical/median animals** | **Typical/median knees** | **Key implication** |
| --- | --- | --- | --- | --- | --- | --- |
| HE_histology_score | 12 | 12 | 12 | 9 | 17 | Intact coverage is limited to one sample/animal; histology is supportive/exploratory. |
| IHC_histomorphometry | 28 | 28 | 28 | 10 | 18 | IHC uses sample-level means and exploratory mixed-model screens; interpretation requires FDR context. |
| MicroCT_cortical | 21 | 21 | 21 | 12 | 23 | Cortical/surface outcomes have broader coverage but are not the primary structural domain. |
| MicroCT_surface | 5 | 5 | 5 | 12 | 23 | Surface metrics are exploratory structural readouts. |
| MicroCT_trabecular | 48 | 48 | varies | 5-10 | 6-17 | Only 23 trabecular endpoints match the full trabecular coverage pattern; Percent Trabecular Bone Volume used 10 animals/17 knees. |

*Source table: endpoint_specific_n_audit.csv. Full endpoint-by-endpoint modelability is retained in the machine-readable audit outputs.*

**Supplementary Table S4.** Primary endpoint and selected manuscript-interpreted tissue results.

| **Endpoint** | **Contrast** | **Estimate (95% CI)** | **P value** | **Family q** | **Global q** | **N animals/knees** | **Interpretation** |
| --- | --- | --- | --- | --- | --- | --- | --- |
| Percent Trabecular Bone Volume | Mnx+HA+P6 vs Mnx+HA | +1.52 (-2.22 to 5.26) | 0.427 | 0.762 | 0.909 | 10/17 | Primary structural contrast; no robust added P6 benefit. |
| Percent Trabecular Bone Volume | Mnx+HA vs Mnx | +3.23 (-1.39 to 7.85) | 0.170 | 0.639 | 0.909 | 10/17 | Treatment-versus-injury pattern; not correction-significant. |
| Percent Trabecular Bone Volume | Mnx+HA+P6 vs Mnx | +4.75 (0.13 to 9.37) | 0.044 | 0.298 | 0.515 | 10/17 | Nominal treatment-versus-injury pattern only. |
| Summary cartilage score | Mnx+HA+P6 vs Mnx+HA | +0.11 (-0.20 to 0.43) | 0.481 | 0.936 | 0.909 | 9/17 | No histological added P6 benefit. |
| Summary subchondral area score | Mnx+HA+P6 vs Mnx+HA | -0.21 (-0.70 to 0.27) | 0.391 | 0.936 | 0.909 | 9/17 | No direct added P6 effect. |
| Summary subchondral area score | Mnx+HA vs Mnx | -0.81 (-1.40 to -0.23) | 0.007 | 0.160 | 0.116 | 9/17 | Nominal/supportive treatment-versus-injury pattern. |
| Summary subchondral area score | Mnx+HA+P6 vs Mnx | -1.02 (-1.61 to -0.44) | <0.001 | 0.045 | 0.017 | 9/17 | Supportive injury-reference signal; does not establish added P6 over HA. |

*Source table: primary_key_secondary_results.csv. Lower histological scores are favorable for the injury-oriented H&E summary scores.*

**Supplementary Table S5. s**ample-level IHC/histochemical mean analysis summary.

| **Analysis level** | **Result** | **Interpretation** |
| --- | --- | --- |
| Scope | 28 sample-level IHC/histochemical mean endpoints. | Exploratory/supportive tissue-pattern analysis. |
| Omnibus group testing | No IHC/histochemical mean endpoint survived FDR correction. | No robust group-level IHC/histochemical effect in the strict mean analysis. |
| Primary contrast | No Mnx+HA+P6-versus-Mnx+HA mean endpoint reached nominal significance. | Does not support peptide-specific superiority. |
| Smallest primary-contrast p value | Cartilage - Dark Sirius Red: +10.18 percentage points; p = 0.114. | Trend only; not nominally significant. |
| Only nominal pairwise p < 0.05 across all group comparisons | Cartilage total cartilaginous tissue by Movat: Mnx+HA+P6 > Mnx, +7.71 percentage points; p = 0.043; not FDR-significant. | Exploratory treatment-versus-injury pattern only. |

*Source: IHC_sample_level_mean_analysis.csv and IHC_sample_level_mean_descriptives.csv.*

**Supplementary Table S6.** IHC source reconciliation and reason for apparent FDR mismatch.

| **Analysis source** | **Analysis level** | **Main finding** | **How it is interpreted** |
| --- | --- | --- | --- |
| IHC_sample_level_mean_analysis.csv | Strict Prism-updated sample-level means | No endpoint survives FDR; no nominal Mnx+HA+P6-versus-Mnx+HA IHC endpoint. | Primary IHC mean interpretation; correction-negative. |
| final_LMM_LM_results.csv filtered to IHC_histomorphometry | Animal/knee-level LMM/LM histomorphometry screen | Dark Picrosirius Red Mnx+HA+P6 vs Mnx+HA: +11.69; p = 0.00032; family q = 0.0537; global q = 0.0106. | Exploratory mixed-model screen; globally FDR-supported but narrowly non-significant after IHC-family correction. |
| Manuscript interpretation | Integrated evidence hierarchy | MicroCT is the primary modality; histology and IHC are supportive/exploratory. | The Dark Picrosirius Red signal is reported but does not upgrade the conclusion to peptide-specific superiority. |

*The apparent mismatch is caused by different analysis levels and FDR families. The manuscript explicitly separates the strict sample-level mean analysis from the broader exploratory mixed-model screen.*

**Supplementary Table S7. Exploratory IHC/histomorphometry mixed-model signals retained for context.**

| **Endpoint** | **Contrast** | **Estimate (95% CI)** | **P value** | **Family q** | **Global q** | **Evidence class** | **N animals/knees** | **Interpretation** |
| --- | --- | --- | --- | --- | --- | --- | --- | --- |
| Dark Picrosirius Red | Mnx+HA+P6 vs Mnx+HA | 11.69 (5.32 to 18.06) | 0.00032 | 0.0537 | 0.0106 | Global FDR < 0.05; IHC-family q = 0.0537 | 10/18 | Exploratory collagen-associated matrix signal; globally FDR-supported but narrowly non-significant after IHC-family FDR correction. |
| Dark Picrosirius Red | Mnx+HA+P6 vs Mnx | 11.17 (3.54 to 18.80) | 0.004 | 0.321 | 0.104 | Nominal p < 0.05 | 10/18 | Exploratory treatment-versus-injury signal only. |
| Dark Picrosirius Red | Mnx+HA+P6 vs Intact | 20.69 (6.01 to 35.37) | 0.006 | 0.321 | 0.140 | Nominal p < 0.05 | 10/18 | Baseline-reference contrast; exploratory only. |
| Bone Safranin O | Mnx+HA vs Mnx | 9.43 (1.38 to 17.47) | 0.022 | 0.908 | 0.361 | Nominal p < 0.05 | 10/18 | Exploratory matrix-pattern signal; not correction-significant. |
| cartilage Movat | Mnx+HA vs Mnx | -14.98 (-29.40 to -0.55) | 0.042 | 0.931 | 0.552 | Nominal p < 0.05 | 10/18 | Exploratory image-derived signal; not correction-significant. |

*Source: final_LMM_LM_results.csv filtered to endpoint_family == IHC_histomorphometry.*

**Supplementary Table S8.** Sensitivity-analysis summary.

| **Analysis** | **Purpose** | **Main interpretation** |
| --- | --- | --- |
| Ordinal H&E cumulative-link sensitivity | Assesses ordinal histology scores without treating score steps as fully continuous. | No ordinal sensitivity result changed the primary conclusion. |
| IHC beta/fractional sensitivity | Checks bounded 0-100% IHC/histochemical percentages. | Supports exploratory interpretation; no robust peptide-specific superiority. |
| Heteroscedastic GLS sensitivity | Checks group-specific residual variance where relevant. | No decisive upgrade of the primary contrast. |
| Animal-cluster bootstrap and animal-level permutation | Checks non-parametric uncertainty at sheep/animal level. | Supports endpoint-specific caution and avoids image/section-level pseudoreplication. |
| Leave-one-animal-out influence | Checks whether signals are driven by one animal. | Useful for robustness; no single-animal result justifies stronger claims. |
| Normal-approximation Bayesian microCT sensitivity | Quantifies uncertainty for key microCT endpoints. | Supports uncertainty framing rather than confirmatory superiority. |
| Direction-aligned repair score | Exploratory integrated structural/tissue pattern summary. | Useful as a descriptive pattern analysis only. |
| Power/precision simulation | Estimates future confirmatory design requirements. | Used to frame future work, not current adequacy. |

*Sources: HE_ordinal_cumulative_link_sensitivity.csv, IHC_beta_fractional_sensitivity.csv, heteroscedastic_GLS_sensitivity.csv, bootstrap_leave_one_out_permutation_robustness.csv, direction_aligned_repair_score.csv, and simulation outputs.*

**Supplementary Table S9.** Exploratory integrated repair-score contrasts.

| **Contrast** | **Estimate** | **95% CI lower** | **95% CI upper** | **P value** | **Interpretation** |
| --- | --- | --- | --- | --- | --- |
| Mnx+HA+P6 vs Mnx+HA | 0.13 | -0.92 | 1.18 | 0.799 | No integrated-score evidence of added P6 benefit. |
| Mnx+HA vs Mnx | positive direction not used for confirmatory inference | NA | NA | NA | Exploratory only. |
| Mnx+HA+P6 vs Mnx | positive direction not used for confirmatory inference | NA | NA | NA | Exploratory only. |

*Source: direction_aligned_repair_score_results.csv. The repair score is exploratory and should not replace endpoint-level inference.*

**Supplementary Table S10.** Sample-size and precision simulation summary for future confirmatory work.

| **Endpoint** | **Purpose** | **Interpretation for future study design** |
| --- | --- | --- |
| Percent Trabecular Bone Volume | Primary structural endpoint for added-value contrast. | Observed Mnx+HA+P6-versus-Mnx+HA effect is small/imprecise; substantially larger and powered confirmatory designs are required. |
| Trabecular separation | Key secondary structural endpoint. | Potentially useful for treatment-versus-injury patterns, but not confirmatory for added P6 benefit in the pilot. |
| Trabecular thickness/number | Key secondary structural endpoints. | Useful for exploratory architecture, but requires prespecified handling and sufficient coverage. |
| Subchondral summary score | Supportive histological endpoint. | Can inform tissue context but should not stand alone as a primary efficacy endpoint. |

*Source: simulation_power_precision_results.csv. Simulations are used only to plan future confirmatory work.*

# Additional compact statistical support tables

The following compact tables were added to keep the Word supplement readable while preserving full reproducibility in the machine-readable CSV/XLSX outputs. Canonical key microCT structural results are summarized in Supplementary Table S11, primary-contrast robustness checks in Supplementary Table S12, source selection and unit harmonization in Supplementary Table S13, and the machine-readable output index in Supplementary Table S14. Full LMM/LM, normal-approximation posterior, leave-one-out, exclusion-audit, and simulation tables remain available as data-supplement files rather than being printed in full.

**Supplementary Table S11.** Canonical key microCT structural endpoint results.

| **Endpoint** | **Unit** | **Contrast** | **Estimate (95% CI)** | **P / family q / global q** | **N animals/knees** | **Interpretation** |
| --- | --- | --- | --- | --- | --- | --- |
| Percent Trabecular Bone Volume | % | Mnx+HA+P6 vs Mnx+HA | 1.52 (-2.22 to 5.26) | 0.427 / 0.762 / 0.909 | 10/17 | No robust difference. |
| Percent Trabecular Bone Volume | % | Mnx+HA+P6 vs Mnx | 4.75 (0.13 to 9.37) | 0.044 / 0.298 / 0.515 | 10/17 | Nominal difference only after multiplicity correction. |
| Percent Trabecular Bone Volume | % | Mnx+HA vs Mnx | 3.23 (-1.39 to 7.85) | 0.170 / 0.639 / 0.909 | 10/17 | No robust difference. |
| Trabecular separation | um | Mnx+HA+P6 vs Mnx+HA | 54.11 (29.42 to 78.80) | <0.001 / <0.001 / <0.001 | 6/11 | Higher separation in Mnx+HA+P6; direction does not support added structural benefit over Mnx+HA. |
| Trabecular separation | um | Mnx+HA+P6 vs Mnx | 12.24 (-5.56 to 30.05) | 0.178 / 0.639 / 0.909 | 6/11 | No robust difference. |
| Trabecular separation | um | Mnx+HA vs Mnx | -41.87 (-59.40 to -24.34) | <0.001 / <0.001 / <0.001 | 6/11 | Global FDR <0.05; interpret with endpoint direction. |
| Trabecular thickness | um | Mnx+HA+P6 vs Mnx+HA | -0.56 (-26.10 to 24.97) | 0.965 / 0.998 / 1.000 | 6/11 | No robust difference. |
| Trabecular thickness | um | Mnx+HA+P6 vs Mnx | 15.12 (-3.46 to 33.70) | 0.111 / 0.524 / 0.835 | 6/11 | No robust difference. |
| Trabecular thickness | um | Mnx+HA vs Mnx | 15.68 (-2.48 to 33.85) | 0.091 / 0.462 / 0.753 | 6/11 | No robust difference. |
| Trabecular number | 1/um | Mnx+HA+P6 vs Mnx+HA | -1.95e-04 (-2.48e-04 to -1.41e-04) | <0.001 / <0.001 / <0.001 | 6/11 | Lower trabecular number in Mnx+HA+P6; direction does not support added benefit over Mnx+HA. |
| Trabecular number | 1/um | Mnx+HA+P6 vs Mnx | -8.97e-05 (-1.28e-04 to -5.10e-05) | <0.001 / <0.001 / <0.001 | 6/11 | Global FDR <0.05; interpret with endpoint direction. |
| Trabecular number | 1/um | Mnx+HA vs Mnx | 1.05e-04 (6.69e-05 to 1.43e-04) | <0.001 / <0.001 / <0.001 | 6/11 | Global FDR <0.05; interpret with endpoint direction. |

**Supplementary Table S12.** Robustness and sensitivity summary for the primary Mnx+HA+P6 versus Mnx+HA contrast.

| **Endpoint** | **Contrast** | **Main model estimate (95% CI)** | **Cluster-bootstrap CI** | **Permutation P** | **Post. prob. favorable** | **Robustness interpretation** |
| --- | --- | --- | --- | --- | --- | --- |
| Percent Trabecular Bone Volume | Mnx+HA+P6 vs Mnx+HA | 1.52 (-2.22 to 5.26) | -10.09 to 4.40 | 0.799 | 0.787 | No added-benefit signal. |
| Trabecular separation | Mnx+HA+P6 vs Mnx+HA | 54.11 (29.42 to 78.80) | 93.23 to 106.50 | 0.348 | 1.000 | Model signal; permutation not supportive. |
| Trabecular thickness | Mnx+HA+P6 vs Mnx+HA | -0.56 (-26.10 to 24.97) | -11.10 to 1.97 | 1.000 | 0.483 | No added-benefit signal. |
| Trabecular number | Mnx+HA+P6 vs Mnx+HA | -1.95e-04 (-2.48e-04 to -1.41e-04) | -3.48e-04 to -2.89e-04 | 0.334 | <0.001 | Model signal; permutation not supportive. |

**Supplementary Table S13.** Source selection, exclusions, and unit harmonization.

| **Data component** | **Issue addressed** | **Retained for reader-facing interpretation** | **Excluded or de-emphasized** | **Reason** |
| --- | --- | --- | --- | --- |
| MicroCT trabecular canonical endpoints | Primary/key structural outputs | Percent BV/TV, Tb.Sp, Tb.Th, and Tb.N retained as canonical reader-facing endpoints. | Distribution-bin endpoints were excluded from manuscript inference. | Prevents duplicate/technical bin endpoints from inflating interpretation. |
| MicroCT duplicate unit variants | Unit harmonization | Micrometer-based entries (um and 1/um) retained for Tb.Sp, Tb.Th, and Tb.N reader tables. | Parallel U or 1/U variants retained only in machine-readable output. | Improves readability and avoids duplicating the same biological endpoint. |
| H&E histology | Animal/knee-level mixed analysis | Mapped grading data retained with sheep/animal random-effect structure where repeated knees were present. | Unmapped or noncanonical score variants not emphasized in main interpretation. | Protects the animal/knee experimental unit. |
| IHC/histochemical quantification | Exploratory/supportive analysis | Updated sample-level mean source used for reader-facing exploratory summaries. | Older/duplicated source representations reconciled and not counted twice in global FDR interpretation. | Avoids duplicate mechanistic endpoints and overstatement of nominal signals. |
| Full model and sensitivity outputs | Machine-readable reproducibility files | Full LMM/LM, normal-approximation posterior, leave-one-out, and simulation tables retained as CSV/XLSX. | Not printed in full in the Word supplement. | Provides transparency without creating unreadable tables. |

**Supplementary Table S14.** Machine-readable statistical output index.

| **File or sheet** | **Size** | **Contents** | **Reader-facing handling** |
| --- | --- | --- | --- |
| final_LMM_LM_results.csv | 732 x 28 | Full mixed/linear model contrast output for all modelable endpoints. | Too long for Word; retained in data supplement. |
| Bayesian_microCT_normal_approximation.csv | 492 x 33 | Normal-approximation posterior uncertainty sensitivity for microCT endpoints. | Not full MCMC; summarized only. |
| bootstrap_leave_one_out_permutation_robustness.csv | 69 x 11 | Cluster bootstrap and animal-label permutation checks for key endpoints. | Summarized for key contrasts. |
| leave_one_animal_out_influence.csv | 477 x 6 | Leave-one-animal-out influence analysis. | Use stability summary rather than all rows. |
| endpoint_specific_n_audit.csv | 130 x 16 | Endpoint-level animal/knee coverage and modelability audit. | Coverage summarized in Tables S2-S3. |
| exclusion_audit.csv | 295 x 42 | Detailed row/source exclusion and noncanonical endpoint audit. | Summarized in source/unit harmonization table. |
| sample_size_precision_simulation.csv | 266 x 7 | Future-study sample-size and precision simulations. | Condensed in Table S10. |
| Smart_Medicine_R460_consolidated_tables.xlsx | 12 sheets | Workbook collecting primary machine-readable analysis tables. | Recommended source for reviewers requiring complete outputs. |
